# A General HIV Incidence Inference Scheme Based on Likelihood of Individual Level Data and a Population Renewal Equation

**DOI:** 10.1371/journal.pone.0044377

**Published:** 2012-09-12

**Authors:** Guy Severin Mahiane, Rachid Ouifki, Hilmarie Brand, Wim Delva, Alex Welte

**Affiliations:** 1 South African Centre for Epidemiological Modelling and Analysis, Stellenbosch University, Stellenbosch, South Africa; 2 International Centre for Reproductive Health, Gent University, Gent, Belgium; University of Hong Kong, Hong Kong

## Abstract

We derive a new method to estimate the age specific incidence of an infection with a differential mortality, using individual level infection status data from successive surveys. The method consists of a) an SI-type model to express the incidence rate in terms of the prevalence and its derivatives as well as the difference in mortality rate, and b) a maximum likelihood approach to estimate the prevalence and its derivatives. Estimates can in principle be obtained for any chosen age and time, and no particular assumptions are made about the epidemiological or demographic context. This is in contrast with earlier methods for estimating incidence from prevalence data, which work with aggregated data, and the aggregated effect of demographic and epidemiological rates over the time interval between prevalence surveys. Numerical simulation of HIV epidemics, under the presumption of known excess mortality due to infection, shows improved control of bias and variance, compared to previous methods. Our analysis motivates for a) effort to be applied to obtain accurate estimates of excess mortality rates as a function of age and time among HIV infected individuals and b) use of individual level rather than aggregated data in order to estimate HIV incidence rates at times between two prevalence surveys.

## Introduction

Accurate HIV incidence estimates are essential for determining public health priorities and assessing the impact of interventions in order to monitor the HIV epidemic, but because HIV infections are silent events, estimation of HIV incidence is difficult [1]. The most direct approach to estimating HIV incidence is through observational studies, in which subjects are periodically monitored for HIV infection, but such studies are time consuming and expensive, and may provide biased estimates. Another approach that has attracted considerable attention [2]–[6] is the use of HIV tests that can discriminate recent infections, based on a single specimen. The theoretical underpinnings of this approach have recently been put on a sound footing under general assumptions [7] but in practice the tests themselves, despite recent progress [8], [9] have not yet evolved to the point where they yield consistently informative estimates [10]–[13]. Numerous ways have also been proposed to relate incidence to (possibly multiple) cross-sectional measures of prevalence [14]–[25]. Most of these use age-specific prevalence from only one survey [14], [16]–[18], [22], others rely on long time series [15], [23], [26].

Among the methods that use multiple unlinked cross-sectional surveys, three methods warrant a closer look. They are variations on the theme of interpreting two successive age structured prevalence surveys into estimates of parameters of a population dynamic model which aggregates the effects of infection and mortality over the time between the two surveys. Brunet and Struchiner [24] derived a fundamental formula, which also forms the core of the present work, expressing the incidence as a function of excess mortality rates and prevalence, in an age and time structured susceptible-infected (SI) model. Their method of estimating incidence is based on smoothing data from repeated age structured prevalence surveys and estimates of cumulative excess mortality rates. Hallett et al. [19] proposed a variation on this theme, now commonly cited, and used to estimate HIV incidence in sub-Saharan African settings [19], [27], [28], which is based on the assumption of constant incidence within age bins, and during the period between two surveys [19]. Brookmeyer and Konikoff [29] proposed yet another variation, which parameterises the different mortalities of the infected and susceptible population into a survival ratio.

In this paper, we propose an alternative approach based on serostatus observations over a range of times and ages, such as for example multiple age-structured prevalence surveys. It entails viewing infection, ageing, and death as continuous processes at any point in time, and merely using available serostatus data to obtain the best possible estimate of the rate of change of prevalence at an age and time of interest, we circumvent the need to calculate artefactual aggregated summary parameters. Thus, the fundamental equation first used for this purpose by Brunet and Struchiner [24] can be applied more systematically. We use a simulated epidemic to assess applicability to the case of HIV. The special case of a pure birth cohort is also invoked, to demonstrate subtleties of the assumptions and approximations, and to facilitate comparison of methodological choices. The performance of the introduced estimator is compared to those proposed by Brunet and Struchiner [24], Hallet et al. [19] and Brookmeyer and Konikoff [29].

## Methods

### General Framework

Consider, in an age structured population, a non remissible infection which induces a difference in mortality rate. This can be embodied in an SI-type model (See Text S1, Appendix A) with a ‘*force of infection*’ or incidence *rate*, i.e. as the number of infections *per susceptible person-time*, 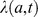. The idea that people of age *a* at time *t* become the people of age 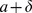 after a period of duration 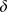, to the extent that they survive, is a core tenet of what is usually referred to as ‘*population renewal’*. Making no assumptions about *transmission dynamics*, and merely parameterising the process of ageing, infection, and death, through *rates*, leads, as demonstrated by Brunet and Struchiner [24], [30], to
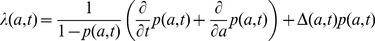
(1)where 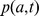 and 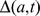 represent the prevalence and excess mortality rate of the infected individuals respectively. In the SI model, people aged *a* at time *t* move out of the susceptible population at the total rate 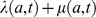, where 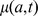 is the background mortality rate. Individuals who become infected enter the infected class, and people of the infected class move out of that class at the rate 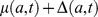. In reality, individuals will have particular infection times, and it is possible to consider further discriminating the infected population by ‘*time since infection*’ (sometimes called ‘*duration*’) and to explicitly have mortality rates depend on this additional parameter. The simplified model does not disregard this fact, but requires that the excess mortality parameter properly accounts for it. The point behind the simplification is to map the model onto observable data, and time since infection is not observable – if it were, we would have simple ways of estimating recent incidence rate from the distribution of times since infection.

The model equations treat all population counts, and hence prevalence, as smooth functions of age and time. Initially, this is conceived as a closed population, but in a more general setting, where the population is subject to migration, 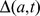 can still be defined, namely as the difference in *net attrition rate* between the infected and uninfected individuals. This is discussed in Text S2 (Section IV).

Equation (1) shows that only the *difference* in mortality rate between the infected and uninfected population is needed, to turn knowledge of prevalence into knowledge about incidence. In practice, estimates of the ‘*excess*’ mortality rate may be inextricably linked to estimates of the background mortality rates, and the nuances of how these estimates are to be obtained are beyond the present work. Brunet and Struchiner [24] suggested using the integral form of equation (1) to estimate a ‘*locally averaged incidence*’, whereas we focus on the instantaneous incidence. In the remainder of this paper, we develop a method for processing prevalence data, and do not explore in any detail the methods for estimating the excess mortality rates, 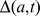.

Formula (1) implies that partial derivatives of the *prevalence*, with respect to age and time, are required to calculate the incidence rate. In practice, only individual HIV serostatus observations are recorded. One way to estimate the derivatives in equation (1) is to use the Maximum Likelihood Estimation (MLE) method. We assume that at least two cross-sectional surveys were conducted between 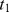 and 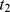 among individuals with ages between 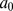 and 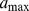. To estimate the derivatives at 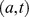 in 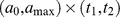, we choose an appropriate 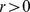 (inclusion window) and for any observed individual *i* aged 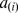 in 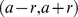 at time 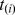, we approximate his/her probability of being infected at that time by keeping the terms of the Taylor expansion which are linear in age and time:
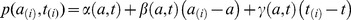
(2)


In the idealised scenario of two cross-sectional surveys instantaneously executed at time 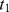 and time 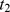, the time when the individual *i* was surveyed, 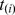, is either 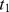 or 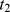, and the individuals are assumed to be independent. The MLE method can thus be applied to the individual serostatus data to yield maximum likelihood estimates of 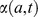, 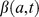 and 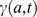, which, as shown in Text S1 (Appendix B), are the estimates of 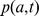, 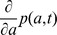, and 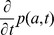, respectively. Inserting these estimates in equation (1) together with the excess mortality rate, 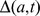, gives an estimate of the incidence rate at 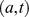. Note that

- this approach is in principle extensible in response to availability of data, so that higher order terms in the Taylor expansion may be kept, reducing bias at the cost of increased variance
- there is no binning of data, and no assumptions about prevalence being piecewise constant or having any other special properties.
- One repeats the whole process for every value of *a* and *t* at which an estimate is to be made, and the inclusion window *r* can in principle be chosen for each value of *(a, t)*
- There is no need for sampling to be population representative in age structure, as there is no averaging being done, which would require a weighting scheme.

### Case of a Birth Cohort

The general ideas of the previous section can be applied to the estimation of incidence rate in a birth cohort. If we consider individuals of the same age at time 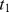 followed up until 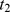, and then assume that the prevalence changes linearly with time, we obtain an explicit expression for our estimator of the incidence rate at any time 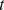 in 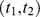 (see Text S1, Appendix C). In the case where 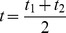, we obtain the 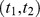-*estimator* given by:
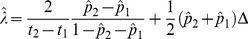
(3)where 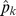 is the maximum likelihood estimation of the prevalence at time 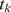
(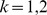), and 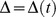 is the excess mortality rate. We show in Text S1 (Appendix B) that equation (3) converges to the true incidence rate when the time gap between the two surveys tends to zero, in which case the variance diverges in practice. There, for the special case of a birth cohort, we give the equation satisfied by the maximum likelihood estimator of the incidence rate which, in general, cannot be solved analytically.

### Comparison to Method of Brunet and Struchiner

Assuming that the excess mortality rate is always positive, Brunet and Struchiner [24] showed that the incidence rate at age *a* can be approximated in the interval 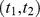 by using the integral form of equation (1). Their formula expresses the incidence rate as a function of the integral of the excess mortality rates (which is assumed to be known) and the prevalence at time 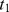 and 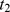. They suggested a weighted smoothing procedure performed by the *lowess* program to create a *smooth surface* from the observed prevalence, and to use this smoothed prevalence in their formula [24]. Brunet and Struchiner’s method differs fundamentally from the approach suggested in this article in that it uses the integral form of equation (1) to estimate a locally averaged incidence, whereas the approach suggested here focuses on the instantaneous incidence. The estimated incidence given by their approach converges to the true incidence rate 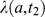 as 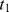 tends to 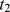, and thus can in principle provide a good approximation of the incidence rate when the prevalence is observed, with high accuracy, very frequently. However, when estimating prevalence shortly after a previous estimate, there may be insufficient precision to resolve the small changes. On the other hand, when the time gap is large, it is less likely that the incidence rate is constant as the method assumes.

### Comparison to Method of Hallett et al

Hallett et al. [19] have developed a method based on simplifying assumptions about population renewal under the pressure of incidence and mortality (closely related approximations to exposure times), the assumption of piecewise constant incidence in age and time bins, and a scheme for ‘*pasting together*’ exposures in the time between two age-binned prevalence distributions. This yields an age binned incidence estimate that is applicable to the time between the two cross-sectional prevalence surveys. Further details to facilitate comparison and evaluation can be found in Text S2 (Section III), where we also calculate asymptotic variances of some closely related estimators for birth cohorts, which are compared to the asymptotic variance of the proposed MLE based incidence estimator, through simulations.

### Comparison to Method of Brookmeyer and Konikoff

Brookmeyer and Konikoff [29] recently proposed aggregating the excess mortality rate of the infected population over the time between two prevalence surveys conducted at 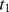 and 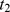. This involved the creation of an alternative parameter: the relative survival rate (R), defined as the ratio of 1) the probability that a person who is HIV infected and alive at time 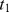 survives to 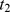, to 2) the corresponding probability in the entire population. An estimator was derived by assuming that: a) the time interval between the two surveys, and/or the incidence, is small; b) the probability that an initially uninfected individual who gets infected during the interval between the surveys, then survives up to the time of the second survey, is the same as the ‘*probability that a person who is uninfected at time 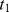 survives to calendar time* 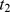
’; and c) the survival probability of an initially uninfected individual is approximately equal to the weighted average of the survival probabilities of an initially uninfected and an initially infected individual. We calculated the exact expressions of the survival probabilities and R by using the SI model (see Section III-1 in Text S2). In the limit that 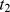 immediately follows 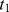, their estimator shall reduce to that of the present work, but as the time between surveys grows, the simplifying assumptions noted above introduce differences.

### Simulations

**Figure 1:**
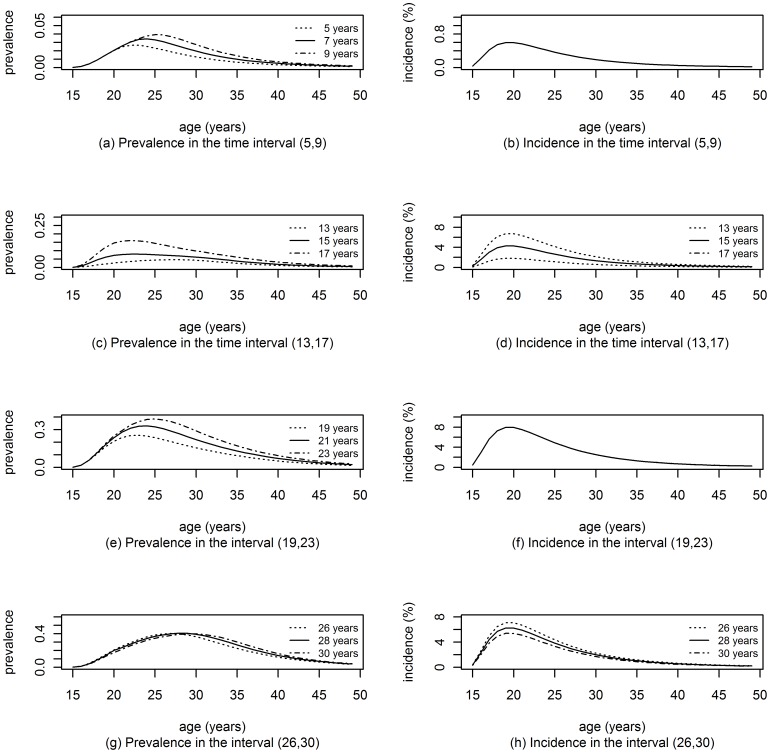
The simulated incidence and prevalence. Simulated age-specific incidence and prevalence at times were the surveys were simulated and at midpoint of intervals of interest.

We simulated an epidemic with four phases; the age specific incidence was constant in time (measured in years) in the first phase (from 0 to 12), increasing in the second (12 to 18), constant in the third (18 to 24) and decreasing in the last phase (24 to 30). Two cross-sectional surveys were simulated in each phase: at times (5, 9), (13, 17), (19, 23) and (26, 30). We used the generated age and time specific prevalence to simulate the infection status of individuals in these surveys (see Text S2, Section II, for further details). Figure 1 illustrates the true age specific prevalence and incidence rate in our simulated population at times where the surveys were conducted and at times where the incidence rate is estimated. The excess mortality rate was specified as a function of time and age. Data from each survey consisted of age and HIV status of 4000 individuals: 200 for each age from 15 to 30 years and 100 for each age from 31 to 49 years. Finally, for each simulation, we estimated 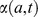, 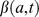 and 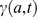 by applying MLE to the simulated data to estimate the simulated incidence at time *t* = 7, 15, 21 and 28. The accuracy and precision of the estimated incidence were investigated by comparing the results of 1000 simulated data sets per scenario, to the input values. The method of Brunet and Struchiner [24] and the method of Hallett et al. [19] were also applied to the simulated data.

In the particular case of a population with a common birth date, we used the closed form of the prevalence (see equation (C2) in Text S1 Appendix C) to simulate the prevalence of an infection with differential mortality in a population at time 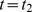 given three parameters: a) the prevalence at the beginning of the observation period (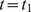); b) the (constant) incidence rate between the two surveys; and c) a constant excess mortality rate. We used the simulated prevalence and the excess mortality rates to estimate the incidence rate using the 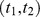-estimator, the estimator proposed by Hallett et al. for birth cohorts (H-estimator) and the estimator proposed by Brookmeyer and Konikoff (B-estimator). This allowed us to measure the bias of these estimators. In the limit of large sample sizes we also compare the standard deviations of these estimators to the standard deviation of the optimal estimator (the numerical solution of equation (C2)).

Sensitivity analysis was performed to assess the effect of the magnitude of incidence rate and the duration between the two surveys on estimates of the incidence rate. Scenarios with non-constant incidence rate were also constructed to investigate the effect of the assumption of constant incidence rate on the estimate of incidence rate. In the latter case, the prevalence was simulated under the hypothesis of varying incidence.

Simulations were run using the **R** programming language [31]. Software in the form of **R** code, together with a sample input data set and complete documentation, is freely available at http://www.incidence-estimation.com/page/r-code-set-for-manuscript-201203.

## Results

**Figure 2:**
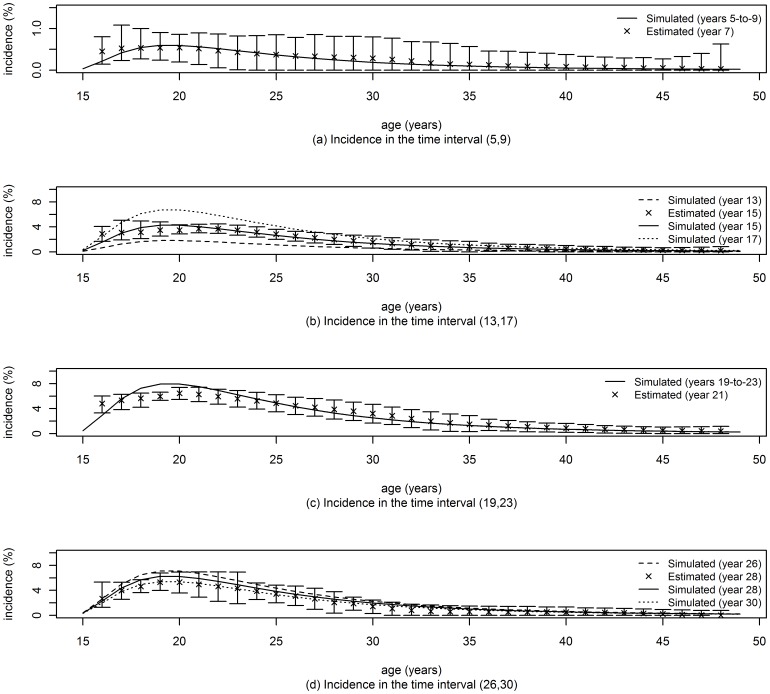
Incidence rates using the MLE approach. The number of replications was 1000 for all the analyses and confidence limits (95% CL) were obtained by the percentile method. The inclusion window 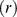 was chosen as follow. a) Period 1, 2 and 3: for times in the interval 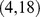, 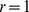 for each age from 15 to 16, 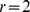 for each age from 17 to 22, 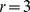 for each age from 23 to 35, and 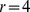 for ages greater than 35. b) Period 4: for times in the interval 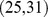, 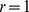 for each age from 15 to 16, 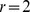 for each age from 17 to 22, 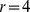 for each age from 23 to 35, and 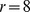 for ages greater than 35.

**Figure 3:**
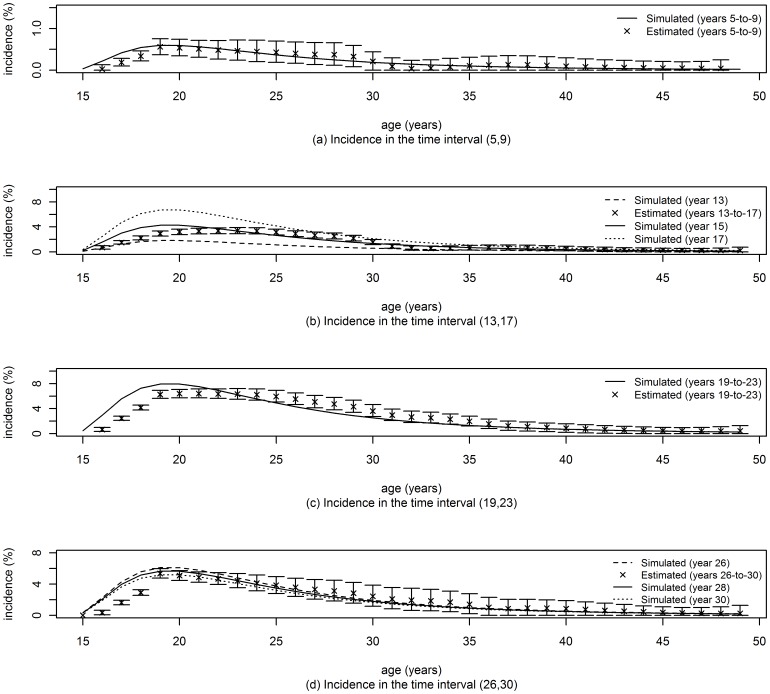
Incidence rates using the approach of Brunet and Struchiner [24]. The number of replications was 1000 for all the analyses and confidence limits (95% CL) were obtained by the percentile method.

**Figure 4:**
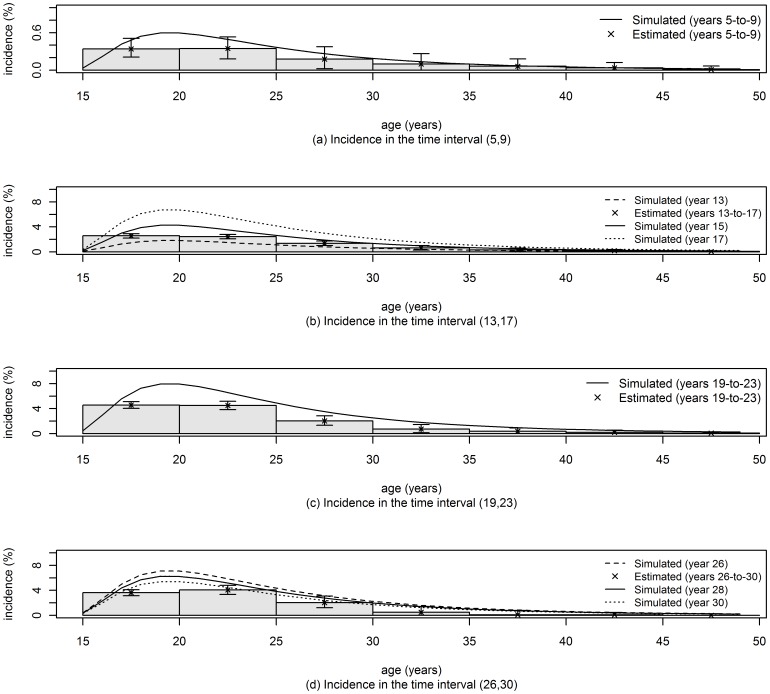
Incidence rates estimated in age bins using the approach of Hallett et al. [19]. The number of replications was 1000 for all the analyses and confidence limits (95% CL) were obtained by the percentile method.

Figures 2, 3 and 4 display the simulated and estimated incidence rates as a function of age for the method developed in this paper, the method of Brunet and Struchiner and the method of Hallet et al. The median of 1000 point estimates is represented by the cross symbol and the error bars represent the central 95 percentile range.

To facilitate direct comparison of the performance of the three methods in figures 2–4, we propose an ad-hoc metric of ‘*success*’ in recovering, through estimation, the incidence rate which was used in the simulation. We define a successful estimate, for the use of two cross sectional prevalence surveys, as one in which the central 95 percentile range of point estimates, based on 1000 randomly generated data sets, includes the mean of the input incidence rates used in the interval between surveys. Then, focusing on the incidence rate in ages up to 30 years, we can see that the ‘*success rate*’, as just defined, for the three methods, across all age and time points reported in figures 2–4, are: 93% (56/60) for the new method of this paper, 53% (32/60) for the method of Brunet and Struchiner, and 33% (4/12) for the method of Hallet et al.

**Figure 5:**
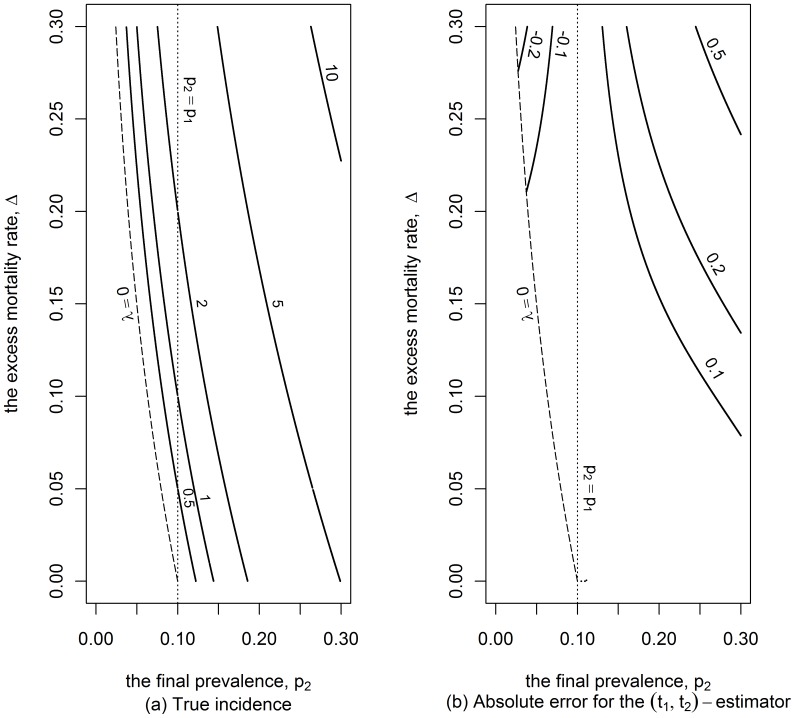
Incidence and absolute error of the 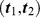-estimator. Contour lines for the true incidence (in percentage) and contour lines for the absolute error (in percentage as well) for the MLE approach in a cohort study in the case where the initial prevalence, 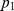 is 0.1 and the time between the two surveys is 5 years.

Figure 5a shows the true incidence rate for a wide range of scenarios where the initial prevalence is 10%. In these scenarios, where both the excess mortality rate and the final prevalence are less than 30%, the incidence rate cannot exceed 10% per annum if the time gap is equal to 5 years. Figure 5b indicates that the absolute error of the 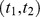-estimator lays in the range −0.002 to 0.005, for the scenarios represented in Figure 5a.

**Figure 6:**
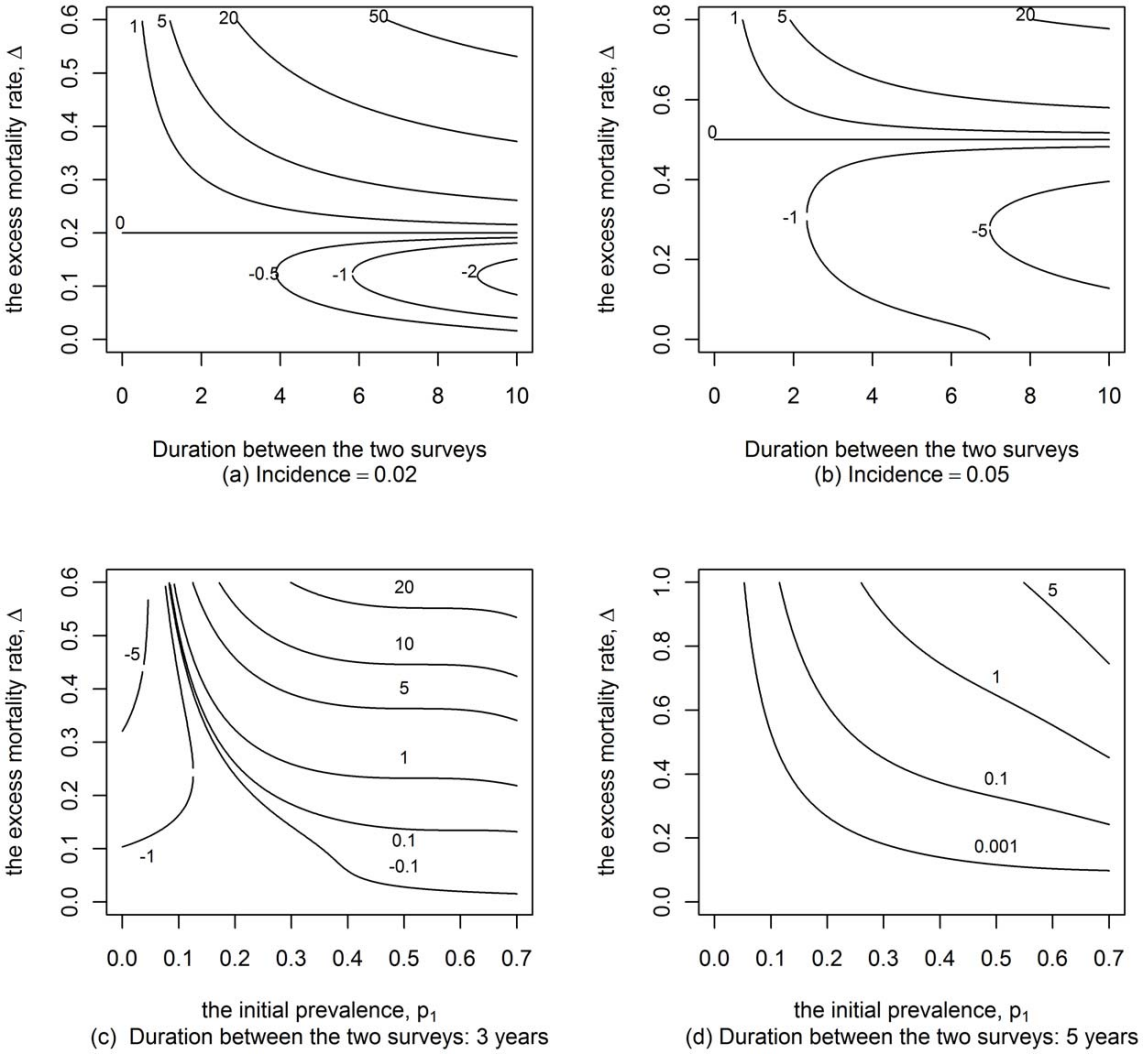
Relative error of the 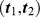-estimator as a function of the initial prevalence. Contour lines for the relative error (in percentage) on the incidence when using the MLE approach in a birth cohort in the case where the duration between the two surveys or the initial prevalence, 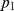 varies.

Additionally we provide a wider view of the ranges of possible relative errors in Figure 6 when the incidence rate or the time gap between the measures of prevalence is fixed. Figures 6a and 6b show that the 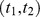-estimator is relatively small when the excess mortality is less than 40%. The error is zero at ‘*endemic equilibrium*’, i.e. when the incidence rate equals excess mortality rate multiplied by the initial prevalence. In the same way, Figures 6c and 6d show that the error is positive and increases for large values of the initial prevalence and excess mortality rates.

Many scenarios were investigated to compare performances of the 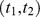-estimator, the H-estimator and the B-estimator in the case of a pure birth cohort. Table 1 contains what we hope is an informative selection of 13 of these scenarios. Note the variability in the parameter values used to generate these scenarios which are provided in the first six rows. The 7^th^, 8^th^ and 9^th^ rows show the values of the three estimates under these conditions. The ratios of the standard deviation of each of the estimators to the standard deviation of the optimal estimator (the numerical solution of equation (C2)) are available in the last three rows. It appears that: a) the H-estimator tends to underestimate the incidence rate, the magnitude of the error being dependent on the excess mortality rate and the initial prevalence; b) reducing the interval between prevalence surveys reduces the bias (scenarios # 8, 10 and 11); c) reducing the excess mortality rate reduces the bias of all the studied estimators (scenario #2); d) the bias of the B-estimator is comparable to the bias of the H-estimator, e) the relative error of the 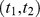-estimator does not exceed 3%; f) the asymptotic standard error of the 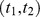-estimator was comparable to the standard error of the optimal estimator of the incidence rate; and g) the asymptotic standard error of the B-estimator was lower than the asymptotic standard error of the 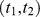-estimator while the asymptotic standard error of the H-estimator was higher than the standard error of the optimal estimator except when the background mortality rate was very small.

**Table 1:** Performances of the estimators of the incidence rates of an infection with a differential mortality in the case of a birth cohort with constant incidence as well as constant background and excess mortalities.

Scenarios	1	2	3	4	5	6	7	8	9	10	11	12	13
**Data**													
*μ*	0.01	0.01	0.01	0.01	0.01	0.01	0.01	0.01	0.01	0.01	0.02	0.02	0.02
*m*	0.1	0.0	0.1	0.1	0.2	0.1	0.1	0.1	0.1	0.1	0.1	0.1	0.2
*t*_2_−*t*_1_	5	5	5	5	5	5	5	3	5	3	3	5	5
*p*_1_	0.1	0.1	0.05	0.2	0.1	0.1	0.1	0.1	0.0	0.1	0.1	0.1	0.1
*p*_2_	0.24	0.30	0.21	0.29	0.19	0.14	0.24	0.19	0.18	0.12	0.19	0.24	0.19
*λ*	0.05	0.05	0.05	0.05	0.05	0.02	0.05	0.05	0.05	0.02	0.05	0.05	0.05
**Incidence estimates**													
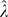	0.049	0.050	0.049	0.050	0.049	0.020	0.049	0.050	0.049	0.020	0.050	0.049	0.049
*λ^H^*	0.039	0.048	0.039	0.040	0.032	0.016	0.039	0.043	0.039	0.017	0.043	0.039	0.031
*λ^B^*	0.038	0.044	0.037	0.039	0.032	0.016	0.038	0.042	0.036	0.017	0.042	0.038	0.032
**Ratio** * **of the ** ***se***													
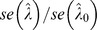	0.97	0.98	0.97	0.98	0.95	0.98	0.97	0.99	0.97	0.99	0.99	0.97	0.94
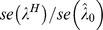	1.16	0.93	1.13	1.20	1.35	4.50	1.16	1.85	1.09	7.83	2.66	2.64	1.80
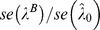	0.69	0.78	0.67	0.71	0.60	0.76	0.69	0.78	0.66	0.84	0.78	0.69	0.60

We used the exact prevalence for the simulations.

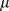
: background mortality rate;

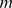
: excess mortality rate;

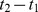
: time between the two surveys;

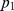
: initial prevalence, at 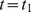;

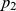
: initial prevalence, at 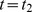;

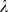
: simulated incidence;

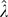
: incidence rate estimated using the 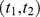-estimator;

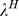
: incidence rate estimated using the H-estimator which was proposed by Hallett et al. [19] for birth cohort;

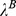
: incidence rate estimated using the B-estimator which was proposed by Brookmeyer and Konikoff. [29];

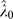
: incidence rate estimated using the optimal estimator obtained by solving the maximum likelihood equations (see equation (C2) in Text S1, Appendix C).

*se*: Standard error; here, the standard deviations were estimated using the delta method; the ratios were calculated under the assumption that the numbers of individuals in the two surveys are the same.

* : Ratio of the *se* to the *se* of the optimal estimator.

Non-constant incidence rate appears to have only a modest impact on our method. In the case of an increasing incidence rate with a constant slope 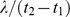 (with 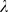 and 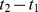 taken from the scenarios described in Table 1), and the incidence rate equals to 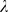 at time 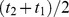, we observed that the 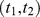-estimator overestimated the incidence rate with a relative error less than 4%. Similarly, under the assumption of decreasing incidence rate with a constant slope of 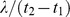 (with 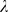 and 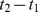 taken from the scenarios described in Table 1), and the incidence rate equal to 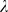 at time 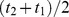, we observed that the 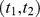-estimator underestimated the incidence rate with a relative error less than 9%.

Additionally, we performed sensitivity analysis for all the scenarios to see how well the 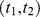
- and the B- and H- estimators performed with an imperfect knowledge of the excess mortality rate (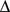). When we increased 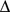 by 20%, the 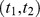-estimator overestimated the incidence rate by no more than 9% while it underestimated the incidence rate by no more than 14% when we decreased 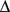 by 20%. In similar conditions, the H-estimator and the B-estimator underestimated the incidence rate with an error varying from 3% to 40%.

## Discussion

The method of estimating incidence rates which we have developed is fundamentally comprised of

- an exact relationship (equation (1)) which formally expresses the force of infection (incidence rate) in terms of the excess mortality rate of the infected state and the current value and rate of change (with respect to age and time) of prevalence; and
- direct estimation of each component of the right hand side of this relationship.

We specifically explored the use of Maximum Likelihood Estimation and a Taylor Series expansion for estimating the required prevalence and its derivatives from individual-level serostatus data, and left open the question of how best to estimate the excess mortality rate in the infected state. This is essentially an instantaneous and individual level formulation of what was cast in aggregated form in previous work, most notably that of Brunet and Struchiner [24], which assumed constant incidence between surveys, and the later work, using additional assumptions, of Hallett et al. [19] and Brookmeyer and Konikoff [29]. Perhaps the fundamental difference in approach is that previous efforts to estimate incidence within this broad paradigm have been based on the idea of a formal solution to a complex dynamical problem, in which a later population state is expressed explicitly in terms of an earlier population state and dynamical rules presumed to have been in effect over the intervening time. This cannot be done in closed form, and indeed is ill posed as dynamical rules can vary in the time interval, hence the use of simplifying assumptions. The present work avoids these difficulties by extracting the estimator directly from the dynamical rules/equations, rather than from a ‘solution’.

A consequence of using formal estimation techniques directly on unaggregated serostatus data is that questions of averaging, and hence of weighting, which complicated bin-based analyses, simply do not arise, and neither does the need for age-representative sampling of study populations. In Text S2 we show how this approach, under additional assumptions, yields key formulas previously used to estimate HIV incidence [18], [23] or more generally, to estimate incidence of infections with differential mortality [20], [25].

Also, estimating incidence rate for a particular age, at a particular point in time, does not require knowledge of the excess mortality rate in an entire interval as in Brunet et al. [24] or the additional assumptions in Hallett et al. [19] and Brookmeyer or Konikoff, such as that incidence rate was small, remained stable in the interval between the surveys and was constant in age bins, or that individuals who become infected between the two surveys survive up to the last survey. Our mathematical analysis (see Section III-1 of Text S2) and numerical simulations showed that these other estimators appear to have significant bias, even with perfect knowledge of prevalence and mortality, sometimes with an inappropriately small formal variance.

Incidence rates can thus, in principle, be estimated for any age, at any time, as long as there is sufficient serostatus and mortality data within a suitable inclusion zone around this point in the age/time domain. The inclusion window can be varied to provide an intuitively straightforward trade-off between variance (less variance with more data) and bias (more bias as data further from the point of interest is interpreted with the low order terms in the Taylor series). While the nature of the trade off is clear enough, and the example calculations suggest the use of serostatus data within 2–5 years of the age of interest (see Figure S4 in Text S2), an *experimenter* in possession of *a single* data set (as opposed to a *simulator* with access to *arbitrarily many,* in addition to the correct answer) cannot directly use this idea to self-diagnose the optimal value of this inclusion criterion. Simulating a number of different scenarios (all of which produce data sufficiently like the real data), will support the choice of robust inclusion criteria.

In this study, we reported 95 percentile ranges of point estimates obtained from a large number of generated data sets. This only makes sense when using simulated data. In a real world context, where serological statuses are observed only once, the bootstrap method described in Text S2 (Section V) can be used to calculate confidence intervals when the sample size is large. When the asymptotic normality is violated, however, the bootstrap method consisting of sampling the individuals with replacement can still be used.

Useful incidence estimates will require sufficiently representative, and numerous, individual HIV serostatus data, and, more problematically, sufficiently accurate and precise excess mortality rate estimates; the challenge being that mortality among HIV infected individuals is related to the incidence in the past, virus sub-type, the availability of Antiretroviral treatment (ART) in the population, etc. Equation (1) suggests that an error in the measure of the excess mortality rate is expected to induce bias in the incidence rate estimate which is of magnitude approximately equal to the bias on the excess mortality rate *multiplied by* the prevalence. Hence, given a reasonable external mortality rate estimate, the bias should be smallest amongst younger people, where the excess mortality rate is low. The problem of error induced by inaccuracy and imprecision of excess mortality rates is inherent to all methodology aiming at estimating HIV incidence from prevalence data, and suggests that this be the focus of substantial additional research, both for the usually primary aim of elucidating the impact of HIV, and for the purpose of stabilising incidence estimates.

Some methodologies for estimating reliable excess mortality rates due to HIV/AIDS have been suggested [32]. Indeed, the estimation of differential mortality is easier for countries where causes of deaths are registered. For developing countries, where deaths records are difficult to access, the verbal autopsy can be used [32]. That method is increasingly used and was shown able to provide reliable mortality rates and detect HIV-related deaths [32]–[34]. What is less clear, and warrants further investigation, is how this data is best analysed to yield differential mortality rates stratified by age and (calendar) time, as opposed to stratified by age and *time since infection*. Furthermore, the increasing availability of ART, will impact the excess mortality associated with HIV infection for years to come.

It is worth noting that our approach can in principle be applied in the more general setting where the population is subject to in- and/or out-migration. In this case, the *excess mortality* is replaced by the *difference*, between infected and uninfected populations, in the ‘*net attrition rates*’, which would then need be known with sufficient accuracy and precision, much as the base case requires knowledge of differential mortality (for further details on accounting for migration/immigration, and the impact of ART, see Section IV of Text S2). Of course, the method is also not restricted solely to the area of HIV incidence estimation and can be used to estimate the incidence rate of any non-remissible infection with differential mortality.

The accurate estimation of HIV incidence will remain a very important issue in public health for many years into the foreseeable future. Given that following cohorts of individuals over time is time consuming, expensive and administratively intensive, and furthermore at risk of yielding biased estimates, a method which allows for the accurate estimation of incidence from cross sectional surveys is of great value. The method proposed in this paper makes no assumptions regarding the epidemiological scenario and in the case of an infinite amount of data, is exact. The only factors which limit the performance of the method are the quality/quantity of the HIV serostatus data, and excess mortality estimates, both of which have been increasing significantly. The three point plan outlined at the beginning of this section provides a break with the artefacts of previous approaches to the use of prevalence and mortality data for estimating HIV incidence, and a systematic framework within which limitations can be better diagnosed as data is analysed.

### Supporting Information

Text S1 contains a simple derivation of Equation (1) the maximum likelihood equations. Derivation of key formulas and technical details of the simulations are given in the Text S2 which also contains an equivalent model and derivation of the equations of some published models to estimate HIV incidence rates from prevalence data. Text S2 also contains description and numerical results of ad hoc methods using aggregated prevalence data to estimate incidence rate as a function of age and ends with a description of the ways to estimate confidence limits of the curve of incidence.

## Supporting Information

Text S1 **Appendix.** Derivation of Equation (1) and description of the maximum likelihood equations
(DOC)

Text S2 **Supplementary Materials for (A General HIV Incidence Inference Scheme Based on Likelihood of Individual Level Data and a Population Renewal Equation).** Derivation of key formulas, details of the simulations, description and numerical results of ad hoc methods using aggregated prevalence data to estimate incidence rate as a function of age and description of the ways to estimate confidence limits of the curve of incidence.
(DOC)
