# Supplementary material for: A General HIV Incidence Inference Scheme Based on Likelihood of Individual Level Data and a Population Renewal Equation
Source: PLoS One. 2012 Sep 12;7(9):e44377. doi: 10.1371/journal.pone.0044377 (PMC3440384; doi:10.1371/journal.pone.0044377)
Supplement: Text S1 — Appendix. Derivation of Equation (1) and description of the maximum likelihood equations (DOC) [file pone.0044377.s001.doc]

**Appendix**

**A. Derivation of formula (1)**

Let $S(a,t)$and $I(a,t)$be the number of individuals susceptible to the infection and the number of infected individuals of age *a* at time *t*, respectively. We model the population by the following SI type model:

$$\left\{\begin{matrix} \frac{\partial}{\partial t}S(a,t)+\frac{\partial}{\partial a}S(a,t)=-\left(\lambda\left(a,t\right)+\mu(a,t)\right)S(a,t) \\ \frac{\partial}{\partial t}I(a,t)+\frac{\partial}{\partial a}I(a,t)=\lambda\left(a,t\right)S(a,t)-\left(\mu(a,t)+\Delta(a,t)\right)I(a,t) \end{matrix}\right. \left(\text{A1}\right)$$

where $\lambda\left(a,t\right)$is the force of infection (or incidence hazard rate or incidence rate), $\mu(a,t)$is the background mortality rate and $\Delta(a,t)$is the difference between the mortality rate in the infected population and the mortality rate in the uninfected population (i.e. *the differential mortality*).

The model described by system (A1) is a completely general model of incidence and mortality rates which makes no assumptions about the underlying epidemiological process. The prevalence of the infection for individuals aged *a* at time *t* is given by$p(a,t)=I(a,t)/\left(I(a,t)+S(a,t)\right)$. Taking the partial of derivatives of $p(a,t)$ and using equation (A1), we obtain:

$$\frac{\partial}{\partial t}p(a,t)+\frac{\partial}{\partial a}p(a,t)=\frac{\lambda\left(a,t\right)S\left(a,t\right)-\left(\mu\left(a,t\right)+\Delta\left(a,t\right)\right)I\left(a,t\right)}{S(a,t)+I(a,t)}+\frac{\mu\left(a,t\right)I\left(a,t\right)}{S\left(a,t\right)+I\left(a,t\right)}+\frac{\Delta\left(a,t\right)I\left(a,t\right)}{\left(S(a,t)+I(a,t)\right)^{2}}$$

which gives equation (1).

**B. MLE of prevalence and its derivatives**

Assume that we observed individuals aged between $a_{0}$ and $a_{\max}$ at times $t_{1}$ and $t_{2}$ ($0\leq t_{1}\leq t_{2}$). To estimate the partial derivatives of the prevalence at a point $\left(a,t\right)$ in$\left(a_{0},a_{\max}\right)\times\left(t_{1},t_{2}\right)$, we proceed by estimating the prevalence in a neighbourhood of$\left(a,t\right)$,$\left(a-r,a+r\right)\times\left(t_{1},t_{2}\right)$, where $r>0$. We assume that there exist$\alpha\left(a,t\right)$, $\beta\left(a,t\right)$and $\gamma\left(a,t\right)$such that, for each individual $\text{i}$observed at time $t_{\left(i\right)}$ in $\left(t_{1},t_{2}\right)$ with age $a_{\left(i\right)}$ in$\left(a-r,a+r\right)$, we have equation (2). We further assume that the observed individuals are independent and, given that the probability that individual $\text{i}$ is infected at time $t_{\left(i\right)}$is$p\left(a_{\left(i\right)},t_{\left(i\right)}\right)$, we estimate $\left(\alpha\left(a,t\right),\beta\left(a,t\right),\gamma\left(a,t\right)\right)$ by maximising the likelihood function

$$L=\prod_{i=1}^{N} p(a_{\left(i\right)},t_{\left(i\right)})^{z_{i}}\left(1-p(a_{\left(i\right)},t_{\left(i\right)})\right)^{1-z_{i}} \left(\text{B1}\right)$$

under the constraint $p\left(b,s\right)$in $\text{(0,1)}$ for all $\left(b,s\right)$ in $\left(a-r,a+r\right)\times\left(t_{1},t_{2}\right)$, where N is the number of individuals $\text{i}$such that $\left(a_{\left(i\right)},t_{\left(i\right)}\right)$belongs to $\left(a-r,a+r\right)\times\left(t_{1},t_{2}\right)$ and $z_{i}$ is the infection status of the individual $\text{i}$ at the time when he was observed ($z_{i}=1$ if the individual is infected, $z_{i}=0$ otherwise).

Since $L$ is smooth with respect to its arguments, we conclude from [1] that the estimated value, , converges to $\left(p(a,t),\frac{\partial}{\partial a}p(a,t),\frac{\partial}{\partial t}p(a,t)\right)$ as N goes to infinity.

**C. Incidence in a birth cohort**

When a birth cohort is followed up over time, the variables, age and time, change in exactly the same way (life line) and hence prevalence in effect becomes a function of only one variable (time/age). If we consider a group of individuals aged *c* at time $t_{1}$ followed up until$t_{2}$ then, at time $t$ in$\left(t_{1},t_{2}\right)$, these individuals will have age$a=c+t-t_{1}$. In this case, $X\left(a,t\right)=X\left(c+t-t_{1}\right):=X\left(t\right)$ (where $X$denotes$S$,$I$,$\Delta$, $\mu$, $p$or $\lambda$) and formula (1) reduces to an ordinary differential equation (ODE):$\lambda\left(t\right)=\frac{1}{1-p\left(t\right)}\frac{d}{dt}p\left(t\right)+\Delta\left(t\right)p\left(t\right)$ Even in this case, the integral form of this equation still involves the cumulative excess mortality and does not allow calculating the incidence rate but its integral. Thus we proceed by using the MLE method to estimate the derivative of the prevalence. The one dimensional analogue of the truncated Taylor series previously introduced is as follow.

We assume that for any observed individual i at time$t_{(i)}$, his/her probability of being infected at that time is given by. Let $N_{1}$ and $N_{2}$ be the number of individuals observed at $t_{1}$ and $t_{2}$, respectively; and let $N_{11}$ and $N_{21}$ be the number of infected individuals at $t_{1}$ and $t_{2}$, respectively. The log-likelihood is given by: . Taking the partial derivative of $\log\left(L\right)$ with respect to $\hat{\alpha}$ and $\hat{\beta}$, we obtain the likelihood equations:

and

Since $\hat{p}(t_{1})=\frac{N_{11}}{N_{1}}$ and $\hat{p}(t_{2})=\frac{N_{21}}{N_{2}}$, the likelihood equations become

and

.

Solving the latter system, we obtain

and

Thus an estimation of the incidence rate at any time is given by:

If we take $t=\frac{t_{1}+t_{2}}{2}$and set and , we obtain equation (3).

**Case where the incidence and mortality rates are constant**

In the case where both the incidence ($\lambda$) and the differential mortality ($\Delta$) are constant, the integral form of the ODE reduces to (C2), for all *t* in$\left(t_{1},t_{2}\right)$:

$$p\left(t\right)=\frac{\left(\lambda-\Delta p\left(t_{1}\right)\right)\exp\left(\left(\lambda-\Delta\right)\left(t-t_{1}\right)\right)-\lambda\left(1-p\left(t_{1}\right)\right)}{\left(\lambda-\Delta p\left(t_{1}\right)\right)\exp\left(\left(\lambda-\Delta\right)\left(t-t_{1}\right)\right)-\Delta\left(1-p\left(t_{1}\right)\right)} \left(\text{C2}\right)$$

A similar equation was obtained in [2]. The problem with (C2) is that, in general, it cannot be solved analytically. However, that equation should be used to estimate the incidence of an infection with differential mortality in a birth cohort when: a) the prevalence was observed twice, b) the differential mortality is known, c) the incidence is constant and d) the cohort is closed. When the prevalence is observed and $\Delta$ is known, solving equation (C2) in $\lambda$gives the maximum likelihood estimate of the incidence rate, by the invariance property of maximum likelihood estimators. As it stands equation (C2) cannot handle the case where$\lambda=\Delta$. For this case see Supporting Information Text S2 (Section I).

**References**

1. Kendall M, Stuard A (1979) The Advanced Theory of Statistics: Charles Greffin & Company Limited.

2. Podgor MJ, Leske MC (1986) Estimating incidence from age-specific prevalence for irreversible diseases with differential mortality. Stat Med 5: 573-578.
