# Supplementary material for: A General HIV Incidence Inference Scheme Based on Likelihood of Individual Level Data and a Population Renewal Equation
Source: PLoS One. 2012 Sep 12;7(9):e44377. doi: 10.1371/journal.pone.0044377 (PMC3440384; doi:10.1371/journal.pone.0044377)
Supplement: Text S2 — Supplementary Materials for (A General HIV Incidence Inference Scheme Based on Likelihood of Individual Level Data and a Population Renewal Equation). Derivation of key formulas, details of the simulations, description and numerical results of ad hoc methods using aggregated prevalence data to estimate incidence rate as a function of age and description of the ways to estimate confidence limits of the curve of incidence. (DOC) [file pone.0044377.s002.doc]

**Supplementary Materials for**

**(A General HIV Incidence Inference Scheme Based on Likelihood of Individual Level Data and a Population Renewal Equation)**

Guy Severin Mahiane^1#^, Rachid Ouifki^1^, Hilmarie Brand^1^, Wim Delva^1, 2^, Alex Welte^1^

^1^ South African Centre for Epidemiological Modelling and Analysis, Stellenbosch University, South Africa

^2^ International Centre for Reproductive Health, Gent University, Belgium

^#^: Corresponding Author

Emails:

SGM: [sgmahiane@sun.ac.za](mailto:sgmahiane@sun.ac.za)

RO: ouifkir@sun.ac.za

HB: hbrand@sun.ac.za

WD: wim.delva@ugent.be

AW: alexwelte@sun.ac.za

**Corresponding author:**

Dr. Guy Severin Mahiane, SACEMA, 19 Jonkershoek Rd, 7600 Stellenbosch, South Africa

Tel: +27 21 808 2589; Fax: +27 21 808 2586

**I. The integral and differential corollaries to population renewal**

We considered a non remissible infection with differential mortality in an age structured population. Let $S(a,t)$and $I(a,t)$be the number of individuals susceptible to the infection and the number of infected individuals of age *a* at time *t*, respectively. We model the population by the following SI type model given by system (A1):

$$\left\{\begin{matrix} \frac{\partial}{\partial t}S(a,t)+\frac{\partial}{\partial a}S(a,t)=-\left(\lambda\left(a,t\right)+\mu(a,t)\right)S(a,t) \\ \frac{\partial}{\partial t}I(a,t)+\frac{\partial}{\partial a}I(a,t)=\lambda\left(a,t\right)S(a,t)-\left(\mu(a,t)+\Delta(a,t)\right)I(a,t) \end{matrix}\right.$$

where $\lambda\left(a,t\right)$ is the force of infection (or incidence hazard rate or incidence rate), $\mu(a,t)$is the background mortality rate and $\Delta(a,t)$is the difference between the mortality rate in the infected population and the mortality rate in the uninfected population (i.e. *the differential mortality*).

System (A1) captures the basic idea that the individuals aged *a* at time *t* die or survive while new people are allowed to enter the population at age 0. Using that system, we relate the incidence rate and the prevalence by (1):

$$\lambda(a,t)=\frac{1}{1-p(a,t)}\left(\frac{\partial}{\partial t}p(a,t)+\frac{\partial}{\partial a}p(a,t)\right)+\Delta(a,t)p(a,t)$$

We showed how equation (1) can be used to obtain an estimate of the MLE of the incidence rate. The approach suggested was illustrated on simulated data. Here, we discuss the usage of the integral form of (1). We also present the model used to simulate our population. We present a system equivalent to (A1) where the excess death rates among infected individuals are given as function of time since infection. Some alternative methods to estimate incidence rate are presented and estimation of confidence intervals are discussed as well.

We use the method of characteristic lines to find the solution (in$p\left(a,t\right)$) of (1). Let$U\left(s\right)=p\left(a\left(s\right),t\left(s\right)\right)$, where $a\left(s\right)=a_{0}+s$and $t\left(s\right)=s_{0}+s$. Then (4) becomes:

$$\frac{d}{ds}U\left(s\right)=\left(\lambda\left(a_{0}+s,t_{0}+s\right)-\Delta\left(a_{0}+s,t_{0}+s\right)U\left(s\right)\right)\left(1-U\left(s\right)\right) \left(\text{I-1}\right)$$

If $\lambda$and $m$are integrable functions, we integrate (I-1) and we obtain:

$U\left(s\right)=1-\frac{J\left(s\right)}{K_{0}+K\left(s\right)} \left(\text{I-2}\right)$with $K_{0}=\frac{1}{1-p\left(a_{0},t_{0}\right)}$, $J\left(s\right)=\exp\left(\int_{0}^{s} \left(\Delta(a_{0}+u,t_{0}+u)-\lambda(a_{0}+u,t_{0}+u)\right)du\right)$ and

$K(s)=\exp\left(\int_{0}^{s} \Delta(a_{0}+u,t_{0}+u)J(u)du\right)$. This leads to:

where $\pi(\alpha,\beta)=\exp\left(\int_{\alpha}^{\beta} \left(\Delta(u,t-\beta+u)-\lambda(u,t-\beta+u)\right)du\right)$ for $0\leq\alpha\leq\beta$.

We can see from formula (I-3) that using the integral form of (1) can only allow estimating the integral of the incidence rate, unless we make additional assumptions (parameterization). Moreover, the integral of the excess mortality rate (thus past excess mortality rate) is also required.

For birth cohort, we can simply come back to the notation$X\left(a,t\right)=X\left(c+t,t\right)=X\left(t\right)$. Then, from (I-3), we obtain:

$p\left(t\right)=\frac{i\left(t\right)}{1-p\left(0\right)+i\left(t\right)} \left(\text{I-4}\right)$where $i\left(t\right)=p\left(0\right)\exp\left(\int_{0}^{t} \left(\lambda\left(u\right)-\Delta\left(u\right)\right)du\right)+\left(1-p(0)\right)\int_{0}^{t} \lambda\left(w\right)\exp\left(\int_{w}^{t} \left(\lambda\left(u\right)-\Delta\left(u\right)\right)du\right)dw$. Further, in the case where$\lambda=\Delta=cte$, we have$i\left(t\right)=p(0)+\left(1-p(0)\right)\int_{0}^{t} \lambda\left(w\right)dw$, and:

$$p(t)=\frac{p(0)+\left(1-p(0)\right)\lambda t}{1+\left(1-p(0)\right)\lambda t} \left(\text{I-5}\right)$$

**II The simulated HIV epidemic**

Simulating the prevalence of HIV in a population using System (A1) in Appendix A requires the incidence rate as function of age and time, the background mortality rate as well as the excess mortality rate among the infected. But the excess mortality rate is also a function of the incidence rate in the past. Thus to simulate a HIV epidemic, controlling the incidence rate, we opted to use the survival after infection which is intrinsically independent of the incidence. This requires a system different from, but equivalent to, the one used to derive equation (1).

**II.1 The population model**

Let us consider a non remissible infection in a closed population. Let $S(a,t)$and $\tilde{I}\left(a,t,w\right)$ denote the respective numbers of susceptible and of infected (at age w) individuals of age *a* at time *t* in that population and let us consider the system:

defined for $\left(a,t,w\right)$ in $R^{3}$ such that $a,t\geq0$, $0\leq w\leq a$, with the boundary conditions:

$$\tilde{I}(a,t,a)=\lambda(a,t)S(a,t) ; S\left(0,t\right)=S_{0}\left(t\right) \left(\text{II-2}\right)$$

and the initial condition:

$$\tilde{I}\left(a,0,w\right)=0 ; S\left(a,0\right)=S_{1}\left(a\right) \left(\text{II-3}\right)$$

In the system (II-1)-(II-2), $\lambda(a,t)\geq0$ is the incidence rate of the considered infection among people aged *a* at time *t*; $\mu(a,t)$is the background mortality rate; $h(a,w)$ is the hazard of dying (due to causes related to the infection) at age *a* given that the infection occurred at age *w*.

We assume that the functions $\lambda,\mu,S_{0},S_{1}$ and h are given and satisfy: for all $u\geq0$, $h(u,\cdot)$ is measurable, for all *x*, $w\mapsto\lambda\left(w,x+w\right)$ is continue, $\mu$ is measurable, $S_{0},S_{1}$are continue, and $\lambda,\mu,S_{0},S_{1}$are non negative.

Now, define the functions $\psi$ and $\phi$by: $\psi\left(a,t\right)=\left(0,t-a\right)$ if$t\geq a$, $\psi\left(a,t\right)=\left(a-t,0\right)$ if $a\geq t$ and $\phi(a,t)=0$ if$t\geq a$, $\phi(a,t)=a-t$ if$a\geq t$. Thus, the analytical solution $\left(S,\tilde{I}\right)()$ of the system (II-1)-(II-2) is obtained by integrating over the life lines (method of characteristic lines):

$$S\left(a,t\right)=S\left(\psi(a,t)\right)\exp\left(-\int_{\phi(a,t)}^{a} \left(\lambda\left(u,t-a+u\right)+\mu\left(u,t-a+u\right)\right)du\right) \left(\text{II-4}\right)$$

$$\tilde{I}(a,t,w)=\lambda(w,t-a+w)S(w,t-a+w)\exp\left(-\int_{w}^{a} \left(h(u,w)+\mu(u,t-a+u)\right)du\right) i\text{f} \phi(a,t)\leq w\leq a \left(\text{II-5}\right)$$

The interpretations of (II-4) and (II-5) are straightforward. Formula (II-4) gives the number of susceptible individuals aged *a* at time *t*. Such individuals were aged *a-t* at time 0 (if$a\geq t$) or they were aged 0 at time *t-a* (if$a\leq t$) and survived to both infection and death due to causes other than the infection up to age *a*. Formula (II-5) gives the number of individuals aged *a* at time *t* who became infected at age *w*. These were infected at time *t-a+w* and survived to death related to the infection and other causes with probabilities $\exp\left(-\int_{w}^{a} h(u,w)du\right)$ and$\exp\left(-\int_{w}^{a} \mu(u)du\right)$, respectively.

Now, applying (II-4) for *(w, t-a+w)*, inserting the expression obtained in (II-5) and integrating to obtain the number of infected aged *a* at time *t* ($I\left(a,t\right)$) we obtain:

$$I\left(a,t\right)=\int_{0}^{a} I\left(a,t,w\right)dw=\Lambda\left(a,t\right)S\left(a,t\right) \left(\text{II-6}\right)$$

where

$$\Lambda(a,t)=\int_{\phi(a,t)}^{a} \exp\left(\int_{w}^{a} \lambda(u,t-a+u)du\right)\lambda(w,t-a+w)\exp\left(-\int_{w}^{a} h(u,w)du\right)dw \left(\text{II-7}\right)$$

Formula (II-7) shows that we just have to know $\lambda$ and $h$ to estimate the prevalence as a function of age and time. In fact if, as in the main text, we denote the prevalence for individuals aged *a* at time *t* by$p\left(a,t\right)$, we have:

$$p\left(a,t\right)=\frac{\Lambda\left(a,t\right)}{1+\Lambda\left(a,t\right)} \left(\text{II-8}\right)$$

Formula (II-8) can be used to obtain simulated prevalence in our theoretical population which in turn was used to simulate the surveys.

**Remark II.1**

Formulas similar to (II-8) were used by Williams et al. [1] and Sakarovitch et al [2]. The formula was presented in the quoted references but not the population model. Moreover, assuming the system (II-1)-(II-2) is not time dependent, which is the same as assuming that the considered functions are not time dependent or that the system is stable, we obtained a formula which looks like formula (I-4) and is similar to what was proposed by Gregson et al. [3].

The following result relates the system (II-1) to the system (A1).

**Figure S1: The HIV epidemic.** Input incidence rate, age distribution of the initial population and average prevalence among sexually active individuals as function of time. The total size of the initial population was 200, 000


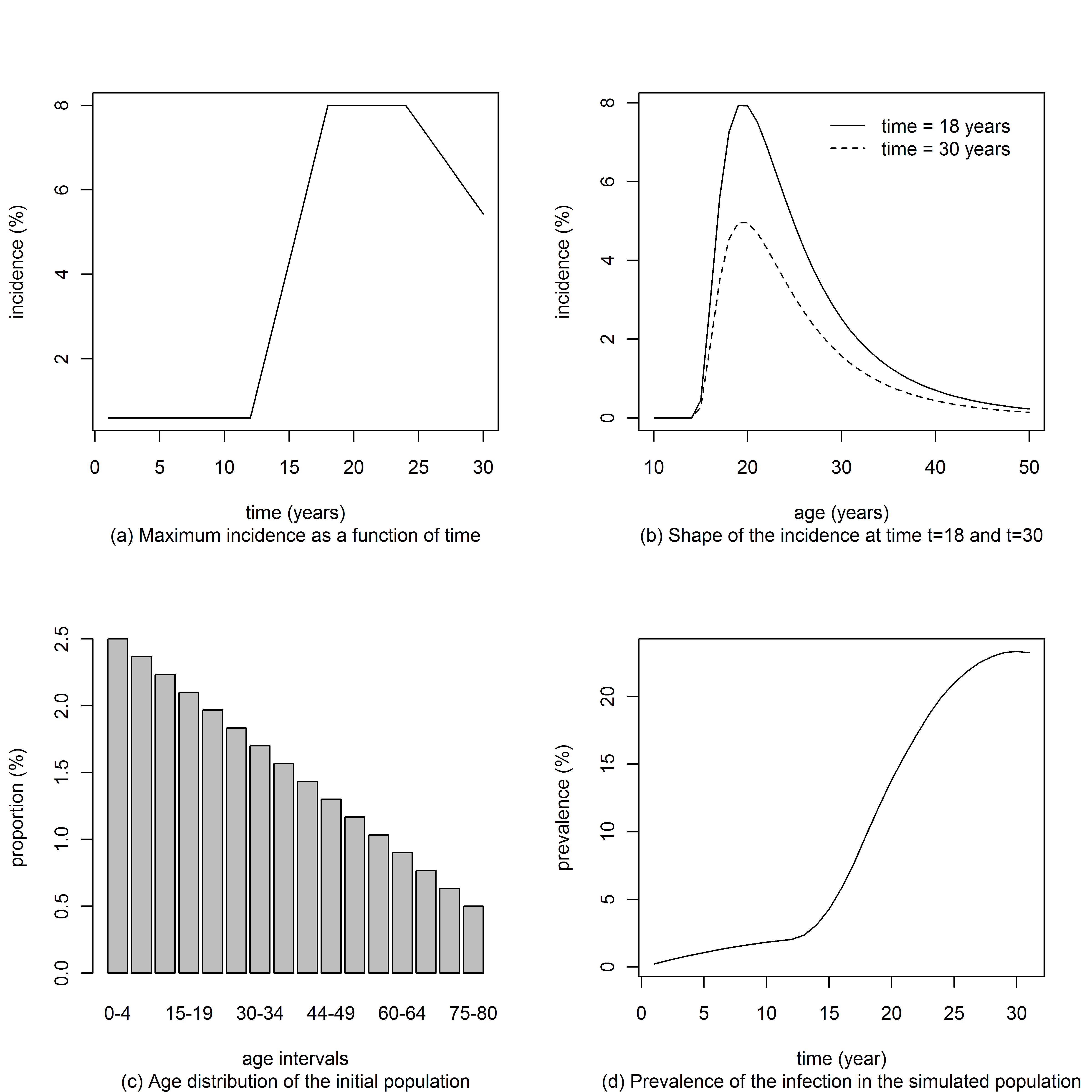


**Remark II.2**

If the incidence rate is differentiable with respect to time, then the couple $\left(S,I\right)$ defined by (II-4) and (II-6) is the solution of the system (A1) with the boundary condition:

$$I\left(0,t\right)=0 ; S\left(0,t\right)=S_{0}\left(t\right) \left(\text{II-9}\right)$$

and the initial condition:

$$I\left(a,0\right)=0 ; S\left(a,0\right)=S_{1}\left(a\right) \left(\text{II-10}\right)$$

with the difference in mortality rate given by:

$$\Delta(a,t)=\frac{\int_{\phi(a,t)}^{a} \exp\left(-\int_{\phi(a,t)}^{w} \lambda(u,t-a+u)du\right)\lambda(w,t-a+w)\exp\left(-\int_{w}^{a} h(u,w)du\right)h(a,w)dw}{\int_{\phi(a,t)}^{a} \exp\left(-\int_{\phi(a,t)}^{w} \lambda(u,t-a+u)du\right)\lambda(w,t-a+w)\exp\left(-\int_{w}^{a} h(u,w)du\right)dw} \left(\text{II-11}\right)$$

**Proof of Remark II.2.**

We just have to show that: $\frac{\partial}{\partial t}I\left(a,t\right)+\frac{\partial}{\partial a}I\left(a,t\right)=\lambda\left(a,t\right)S\left(a,t\right)-\left(\mu\left(a,t\right)+\Delta\left(a,t\right)\right)I\left(a,t\right)$, with $\Delta\left(a,t\right)$ given by (II-11). From (II-6) and since $S$satisfies (II-1) which is identical to the first equation in (A1), we have:

$\frac{\partial}{\partial t}I(a,t)+\frac{\partial}{\partial a}I(a,t)=S(a,t)\left(\frac{\partial}{\partial t}\Lambda(a,t)+\frac{\partial}{\partial a}\Lambda(a,t)-\left(\lambda\left(a,t\right)+\mu(a,t)\right)\Lambda(a,t)\right) \left(\text{II-12}\right)$Now, let $\zeta\left(a,t,w\right):=\exp\left(\int_{w}^{a} \left(\lambda\left(u,t-a+w\right)-h\left(u,w\right)\right)du\right)\lambda\left(w,t-a+w\right)$.

Thus: $\Lambda\left(a,t\right)=\int_{\phi\left(a,t\right)}^{a} \zeta\left(a,t,w\right)dw$. We first observe that $\frac{\partial}{\partial t}\phi(a,t)+\frac{\partial}{\partial a}\phi(a,t)=0$and $\zeta\left(a,t,a\right)=\lambda\left(a,t\right)$. Second, using (II-7), we have:

$\frac{\partial}{\partial a}\Lambda(a,t)=\zeta\left(a,t,a\right)-\zeta\left(a,t,\phi\left(a,t\right)\right)\frac{\partial}{\partial a}\phi\left(a,t\right)+\int_{\phi\left(a,t\right)}^{a} \frac{\partial}{\partial a}\zeta\left(a,t,w\right)dw$ and

$$\frac{\partial}{\partial t}\Lambda(a,t)=-\zeta\left(a,t,\phi\left(a,t\right)\right)\frac{\partial}{\partial t}\phi\left(a,t\right)+\int_{\phi\left(a,t\right)}^{a} \frac{\partial}{\partial t}\zeta\left(a,t,w\right)dw$$

It comes out that:

$$\frac{\partial}{\partial t}\Lambda(a,t)+\frac{\partial}{\partial a}\Lambda(a,t)=\lambda\left(a,t\right)+\int_{\phi\left(a,t\right)}^{a} \left(\frac{\partial}{\partial t}\zeta\left(a,t,w\right)+\frac{\partial}{\partial a}\zeta\left(a,t,w\right)\right)dw \left(\text{II-13}\right)$$

But

$\frac{\partial}{\partial a}\zeta\left(a,t,w\right)=A\exp\left(\int_{w}^{a} \left(\lambda\left(u,t-a+u\right)-h\left(u,w\right)\right)du\right)$ with $A=\left(\left(\lambda\left(a,t\right)+\int_{w}^{a} \frac{\partial}{\partial a}\lambda\left(u,t-a+u\right)du-h\left(a,w\right)\right)\lambda\left(w,t-a+w\right)+\frac{\partial}{\partial a}\lambda\left(w,t-a+w\right)\right)$

and

$\frac{\partial}{\partial t}\zeta\left(a,t,w\right)=B\exp\left(\int_{w}^{a} \left(\lambda\left(u,t-a+u\right)-h\left(u,w\right)\right)du\right)$ with $B=\left(\lambda\left(w,t-a+w\right)\left(\int_{w}^{a} \frac{\partial}{\partial t}\lambda\left(u,t-a+u\right)du\right)+\frac{\partial}{\partial t}\lambda\left(w,t-a+w\right)\right)$.

Then, using the fact that $\frac{\partial}{\partial t}\lambda(u,t-a+u)+\frac{\partial}{\partial a}\lambda(u,t-a+u)=0$, for all u, we obtain:

$$\frac{\partial}{\partial t}\zeta\left(a,t,w\right)+\frac{\partial}{\partial a}\zeta\left(a,t,w\right)=\left(\lambda\left(a,t\right)-h\left(a,w\right)\right)\lambda\left(w,t-a+w\right)\exp\left(\int_{w}^{a} \left(\lambda\left(u,t-a+u\right)-h\left(u,w\right)\right)du\right)$$

Thus:

$$\int_{\phi\left(a,t\right)}^{a} \frac{\partial}{\partial t}\zeta\left(a,t,w\right)+\frac{\partial}{\partial a}\zeta\left(a,t,w\right)dw=\left(\lambda\left(a,t\right)-\Delta\left(a,t\right)\right)\Lambda\left(a,t\right) \left(\text{II-14}\right)$$

Now, inserting (II-14) and (II-13) in (II-12) gives: $\frac{\partial}{\partial t}I\left(a,t\right)+\frac{\partial}{\partial a}I\left(a,t\right)=S\left(a,t\right)\left(\lambda\left(a,t\right)-\left(\mu\left(a,t\right)+\Delta\left(a,t\right)\right)\Lambda\left(a,t\right)\right)$ which, in regard to (II-6), is equivalent to:

$\frac{\partial}{\partial t}I\left(a,t\right)+\frac{\partial}{\partial a}I\left(a,t\right)=\lambda\left(a,t\right)S\left(a,t\right)-\left(\mu\left(a,t\right)+\Delta\left(a,t\right)\right)I\left(a,t\right)$.

**II.2. Epidemiological scenarios**

**HIV infection in the simulated epidemic**

We assumed that incidence rates varied as a function of time and age and was given by (II-15). The age of beginning of sexual activity ($a_{0}$) was taken as an offset and fixed to 14 years. The other parameters, giving the shape and the scale of the distribution, were also fixed: $\beta=2.3$, $\sigma^{2}=0.5$. However, we allowed the normalising factor $R$ to vary as a function of time. Thus the maximum value for the incidence rate varied in four phases. It was constant in the first phase, then increased rapidly in the second phase became constant in the third phase and decreased slowly in the last phase. Figure S1(a) illustrates how the maximum incidence rate varied as a function of time and Figure S1(b) presents the incidence rates as a function of age for *t*=18 and *t*=28 years.

$$\lambda(a,t)=\frac{R(t)}{\sigma(a-a_{0})\sqrt{2\pi}}\exp\left(-\frac{\left(\log(a-a_{0})-\beta\right)^{2}}{2\sigma^{2}}\right) \left(\text{II-15}\right)$$

with

$$R(t)=\sqrt{2\pi}\sigma\exp\left(\beta-\frac{\sigma^{2}}{2}\right)i_{\max}(t) \left(\text{II-16}\right)$$

and

$$i_{\max}(t)=\left\{\begin{matrix} 0.006 & if & 0\leq t\leq12 \\ 0.006+0.123\left(t-12\right) & if & 12\leq t\leq18 \\ 0.08 & if & 18\leq t\leq24 \\ 0.08-0.05\left(t-24\right) & if & 24\leq t\leq31 \end{matrix}\right.$$

**II.3. Generating the data**

We simulated a HIV epidemic following the system (II-1)-(II-2). The system was run for 30 years. As shown in Section B.1, neither the explicit expression of the initial nor the boundary condition of the initial susceptible population is needed in order to obtain the prevalence in that simulated population. However, we generated both the susceptible and infected population and we deduced the prevalence. The total size of the initial population was taken to be 200,000. Its distribution is illustrated in Figure S1(c). The boundary condition, i.e. the new born was the same as the one of the initial population. The hazard of dying as a function of time since infection, for causes related to the infection, *h* was modelled as a Weibull distribution [4], with parameters given in Table S1. These values are from a recent meta-analysis of survival rates in southern and eastern Africa [4].

The input incidence rate was chosen so as to allow it to vary with age and time as it is likely to be in the real world. We simulated incidences rates with log-normal shape with a normalizing factor varying as a function of time as described in Section II.2.

**Table S1:** Parametric Model Estimates of Survival after infection in Southern and Eastern Africa [4]

| Age group | Weibull survival distribution parameters | | |
| --- | --- | --- | --- |
|  | Shape | Scale | Median |
| 15-19 | 2.0 | 16.0 | 13.3 |
| 20-24 | 2.0 | 15.4 | 12.8 |
| 25-29 | 2.0 | 14.1 | 11.7 |
| 30-34 | 2.0 | 12.1 | 10.0 |
| 35-39 | 2.0 | 11.0 | 9.1 |
| 40-44 | 2.0 | 10.1 | 8.4 |
| 45-49 | 2.0 | 7.9 | 6.6 |

We then obtained the *true* prevalence of the infection as a function of age and time in our theoretical population. The curve of the average prevalence in the sexually active population as a function of time is presented in Figure S1(d). We used the prevalence by age to simulate surveys. Given a set of individuals with their ages, we simulated their infection statuses as a function of the true prevalence. The infection status assigned to an individual of age *a* at time *t* was a randomly drawn observation from a Bernouilli distribution with probability of success $p\left(a,t\right)$. This gave the prevalence that would have been observed if a survey was carried out. We used: 200 individuals for each age from 15 to 30 years, and 100 individuals for each age from 31 to 49 years to simulate each survey.

**III. Alternative for estimating incidence from aggregated data**

**III.1. Estimating the partial derivatives of the prevalence using aggregated data**

Often in practice, only aggregated data are available. We assume that prevalence was observed for age bins with centres$a_{k}$, k=1... K, at time$t_{j}$, j=1... J; where K and are J non-zero integers.

**Direct approach**

We approximate the derivatives in (1) using a simple scheme.

$$\frac{\partial p}{\partial a}(a_{k},t_{i})=\frac{1}{2}\left(\frac{p(a_{k+1},t_{j})-p(a_{k},t_{j})}{a_{k+1}-a_{k}}+\frac{p(a_{k},t_{j})-p(a_{k-1},t_{j})}{a_{k}-a_{k-1}}\right) \left(\text{III-1}\right)$$

$$\frac{\partial p}{\partial t}(a_{k},t_{i})=\frac{1}{2}\left(\frac{p(a_{k},t_{j+1})-p(a_{k},t_{j})}{t_{k+1}-t_{k}}+\frac{p(a_{k},t_{j})-p(a_{k},t_{j-1})}{t_{k}-t_{k-1}}\right) \left(\text{III-2}\right)$$

and for boundary points:

$$\frac{\partial p}{\partial a}\left(a_{k},t_{j}\right)=\begin{matrix} \frac{p\left(a_{k+1},t_{j}\right)-p\left(a_{k},t_{j}\right)}{a_{k+1}-a_{k}} & \text{or} & \frac{p\left(a_{k},t_{j}\right)-p\left(a_{k-1},t_{j}\right)}{a_{k}-a_{k-1}} \end{matrix} \left(\text{III-3}\right)$$

$$\frac{\partial p}{\partial t}\left(a_{k},t_{j}\right)=\begin{matrix} \frac{p\left(a_{k},t_{j+1}\right)-p\left(a_{k},t_{j}\right)}{t_{j+1}-t_{j}} & \text{or} & \frac{p\left(a_{k},t_{j}\right)-p\left(a_{k},t_{j-1}\right)}{t_{j}-t_{j-1}} \end{matrix} \left(\text{III-4}\right)$$

Estimating the derivatives of the prevalence as described in (III-1)-(III-4) may yield negative incidence if the distance between the points is large because the prevalence observed is subject to noise. Thus it can be useful to smooth the data before applying these formulas.

**Smoothing the observed prevalence**

It was observed that the prevalence as a function of age can be fitted by a log-normal distribution in some settings. We use:

$$p(a,t_{j})=\frac{R_{j}}{\sqrt{2\pi}\sigma_{j}\left(a-a_{0}\right)}\exp\left(-\frac{1}{\sigma_{j}^{2}}\left(\log\left(a-a_{0}\right)-b\right)^{2}\right) , \text{for} \text{all} j, \text{for} \text{all} a \left(\text{III-5}\right)$$

where $R_{j}$is the normalizing factor and $a_{0}$ is an offset. The parameters$R_{j}$ $\sigma_{j}$ and *b* can be estimated using the binomial error estimate.

The prevalence can also be fitted using cubic splines. In all the cases, the direct approach shall be applied and the estimates inserted in (1) in order to obtain the estimated incidence rates.

**Figure S2:** **Incidence rate and prevalence: using the direct approach on the crude prevalence.**

The surveys were simulated 1000 times and 95% confidence limits were obtained by the percentile method


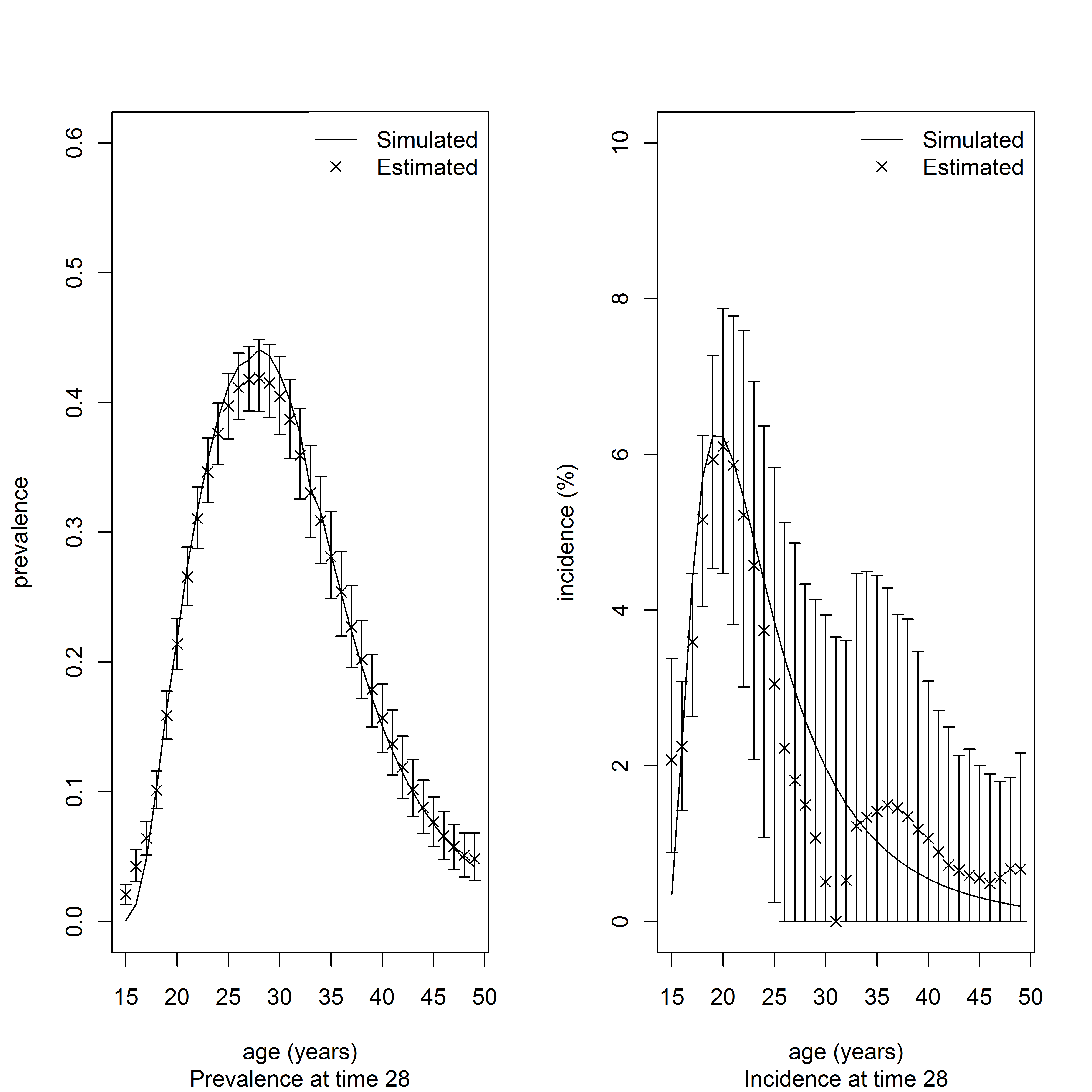


Another approach, which uses smoothed prevalence, was suggested by Brunet and Struchiner [5]. They used (1) to express a “local averaged incidence rate” (for age a in a time between $t_{1}$ and $t_{2}$, $\tilde{\lambda}\left(a,t_{2},t_{1}\right)$) as a function of the prevalence. They found that:

$$\tilde{\lambda}(a,t_{2},t_{1})=\frac{1}{t_{2}-t_{1}}\int_{t_{1}}^{t_{2}} \Delta\left(a-t_{2}+z,z\right)p\left(a-t_{2}+z,z\right)dz+\frac{1}{t_{2}-t_{1}}\log\left(\frac{1-p\left(a-t_{2}+t_{1},t_{1}\right)}{1-p\left(a,t_{2}\right)}\right)$$

Then, using the mean value Theorem, they concluded that there exist $\tau$ in $\left(t_{1},t_{2}\right)$ such that:

$\tilde{\lambda}(a,t_{2},t_{1})=\frac{p\left(a-t_{2}+\tau,\tau\right)}{t_{2}-t_{1}}\int_{t_{1}}^{t_{2}} \Delta\left(a-t_{2}+z,z\right)dz+\frac{1}{t_{2}-t_{1}}\log\left(\frac{1-p\left(a-t_{2}+t_{1},t_{1}\right)}{1-p\left(a,t_{2}\right)}\right)$.

They further suggested to smooth the observed prevalence using the lowess program and took$p\left(a-t_{2}+\tau,\tau\right)=\frac{1}{2}\left(p\left(a-t_{2}+t_{1},t_{2}\right)+p\left(a,t_{1}\right)\right)$. This choice is arbitrary and may induce a large bias in some situations. Moreover the method does not specify the exact time for which the incidence is calculated and may therefore be very imprecise if the time gap between the two measures of the prevalence is long or at best, one has to assume that the incidence is constant.

**Figure S3**. **Incidence rate and prevalence using direct approach on smoothed prevalence.** The number of replications was 1000 for all the analyses. Confidence limits (95% CL) were obtained by the percentile method.


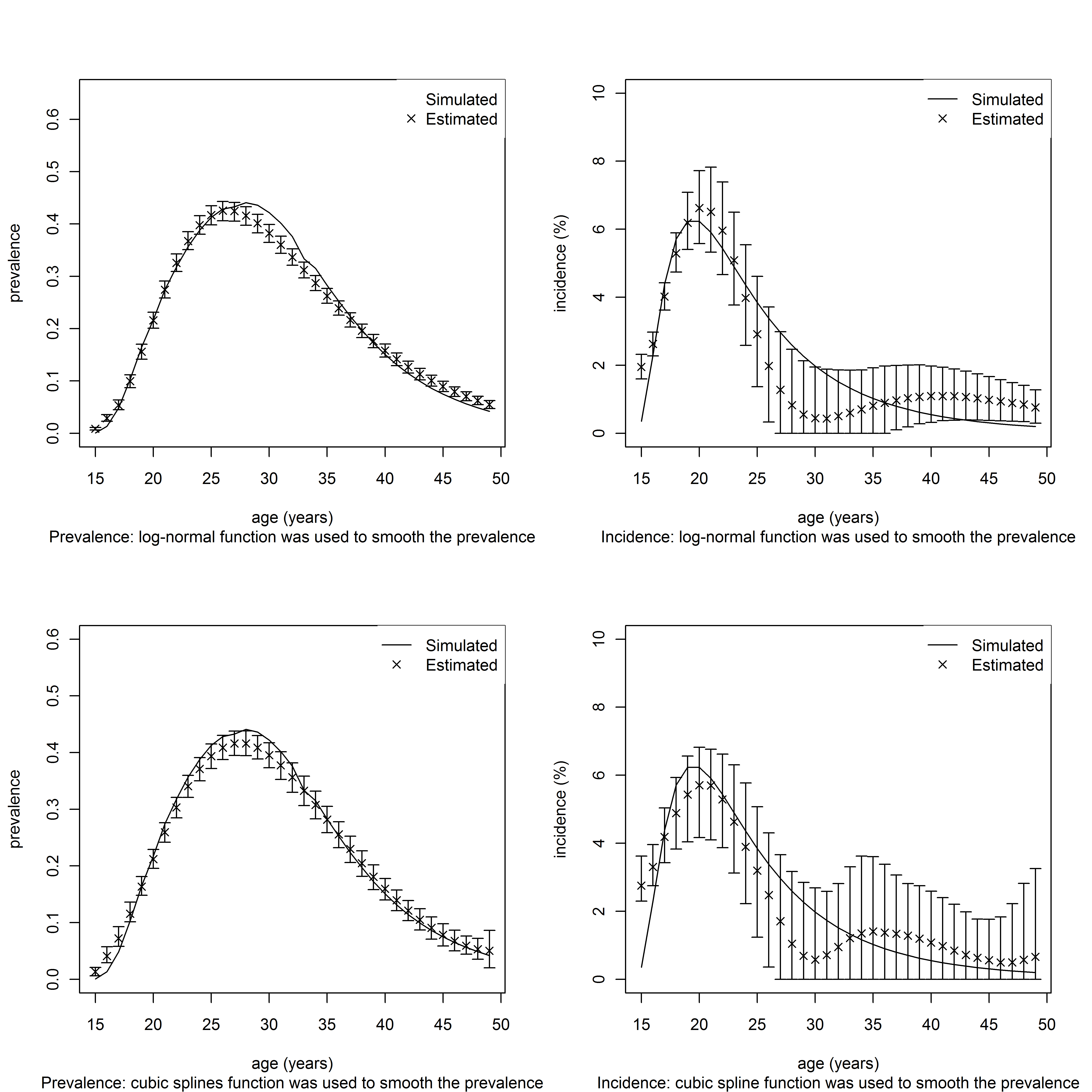


**The synthetic cohort approaches**

Hallett et al. [4] proposed to convert the cohort incidence rate estimates to obtain the conventional age group incidence rate estimates. They suggested estimating the HIV incidence rate in birth cohorts under the assumption that the individuals of the cohort who become infected during the study do not die due to the infection and more, the *H-estimator* was obtained and given by (III-6):

where $\hat{p}_{1}$is the prevalence at $t_{1}$ and $\hat{p}_{2}$is the prevalence at $t_{2}$; approximates the proportionate change in the size of the cohort over the follow-up period, and $\pi=\exp\left(-\left(\mu+\Delta\right)\left(t_{2}-t_{1}\right)\right)$ stands for the probability that people infected at $t_{1}$ survive up to$t_{2}$, with $\mu$ being the background mortality rate and $\Delta$ the excess mortality rate for infected individuals.

The first assumption required to obtain formula (III-6) is that the number of person-years at risk during the two surveys decreases linearly. Following the notations introduced in Section A, this number is $H=\int_{t_{1}}^{t_{2}} S\left(t\right)dt$, where is the solution of $\frac{d}{dt}S\left(t\right)=-\left(\lambda+\mu\right)S\left(t\right)$. Let $T=t_{2}-t_{1}$. Integrating these equations, we found: $H=\frac{S\left(t_{1}\right)-S\left(t_{2}\right)}{\lambda+\mu}$, in contrast to $\tilde{H}=\frac{S\left(t_{1}\right)+S\left(t_{2}\right)}{2}T$ suggested in order to obtain (III-6). Then, using$S\left(t_{2}\right)=S\left(t_{1}\right)\exp\left(-\left(\lambda+\mu\right)T\right)$, we found that the error: $\tilde{H}-H=\frac{S\left(t_{1}\right)}{12}\left(\lambda+\mu\right)^{2}T^{3}+o\left(\left(\lambda+\mu\right)^{2}T^{3}\right)$ depends on the incidence rate, the background mortality rate and the time gap between the two surveys (where *o* is Landrau’s notation). For the approximation to be accurate, $\left(\lambda+\mu\right)^{2}T^{3}$ should be small enough.

More assumptions were needed in order to approximate the proportionate change in size of the cohort: $Q=\left(S\left(t_{2}\right)+I\left(t_{2}\right)\right)/\left(S\left(t_{1}\right)+I\left(t_{1}\right)\right)$, where $I\left(t_{2}\right)=I\left(t_{1}\right)\exp\left(-\left(\mu+\Delta\right)T\right)+\frac{S\left(t_{1}\right)\exp\left(-\mu T\right)}{\Delta-\lambda}\left(\exp\left(-\lambda T\right)-\exp\left(-\Delta T\right)\right)$ was obtained by solving $\frac{d}{dt}I\left(t\right)=\lambda S\left(t\right)-\left(\mu+\Delta\right)I\left(t\right)$ with $S\left(t\right)=S\left(t_{1}\right)\exp\left(-\left(\lambda+\mu\right)\left(t-t_{1}\right)\right)$. The exact expression of that ratio is$Q=p_{1}\exp\left(-\left(\lambda+\Delta\right)T\right)+\left(\frac{\lambda}{\lambda-\Delta}\left(\exp\left(-\Delta T\right)-\exp\left(-\lambda T\right)\right)+\exp\left(-\lambda T\right)\right)\exp\left(-\lambda T\right)\left(1-p_{1}\right)$, which can be compared to the asymptotic value of $\hat{Q}$, $\tilde{Q}$, obtained by replacing $\hat{p}_{1}$ with $p_{1}$. The asymptotic error is then$\tilde{Q}-Q=\left(1-p_{1}\right)\exp\left(-\mu T\right)\left(1-\exp\left(-\lambda T\right)\frac{\lambda}{\lambda-\Delta}\left(\exp\left(-\Delta T\right)-\exp\left(-\lambda T\right)\right)\right)$, which depends on the incidence rate, the excess mortality rate and the duration between the two surveys. Approximating $Q$ with $\tilde{Q}$ implies the epidemiological hypotheses that that the incidence is small and that there is no seroconversion and death due to the infection during the interval between the two surveys

In the same vein, Brookmeyer and Konikoff [6] proposed an estimator to estimate HIV incidence rate from two population-based surveys conducted at two different points in time. Their formula can be derived by using system (A1) applied to a birth cohort and making several assumptions. Indeed, if as in Brookmeyer and Konikoff [6], we denote by $S_{I}$and $S_{U}$ the probability of survival from $t_{1}$to $t_{2}$ of respectively infected and uninfected individuals at time $t_{1}$, then, assuming that the incidence rate is constant, we have:

$$S_{U}=\exp\left(\int_{t_{1}}^{t_{2}} -\left(\mu\left(u\right)+\Delta\left(u\right)\right)du\right)$$

and

$$S_{I}=\exp\left(\int_{t_{1}}^{t_{2}} -\left(\mu\left(u\right)+\lambda\right)du\right)+\lambda\exp\left(\int_{t_{1}}^{t_{2}} -\mu\left(u\right)du\right)\int_{t_{1}}^{t_{2}} \exp\left(\int_{t_{1}}^{w} -\lambda du\right)\exp\left(\int_{w}^{t_{2}} -\Delta\left(u\right)du\right)dw$$

Then the expression of the prevalence at time $t_{2}$ ($p_{2}$) is given by:

where $q_{1}=1-p_{1}$,$S=p_{1}S_{I}+q_{1}S_{U}$,

$$R=\frac{S_{I}}{S} \left(\text{III-7a}\right)$$

and

$$\tilde{S}_{u}=\frac{1}{t_{2}-t_{1}}\left(\exp\left(\int_{t_{1}}^{t_{2}} -\mu\left(u\right)du\right)\int_{t_{1}}^{t_{2}} \exp\left(\int_{t_{1}}^{w} -\lambda du\right)\exp\left(\int_{w}^{t_{2}} -\Delta\left(u\right)du\right)dw\right)$$

Note that Brookmeyer and Konikoff used $S_{U}$ in equation (III-7) instead of $\tilde{S}_{U}$ in order to approximate the prevalence$p_{2}$. The two expressions are equivalent only when $\left(t_{2}-t_{1}\right)\to0$. In fact,

is equivalent to $1+\lambda\left(t_{2}-t_{1}\right)$ when $\left(t_{2}-t_{1}\right)\to0$.

Therefore, if we assume that $S_{u}\approx S$ (as Brookmeyer an Konikoff [6]) a first order approximation (in terms of $\lambda$) of equation (III-7) yields the approximation used by Brookmeyer and Konikoff:$p_{2}=p_{1}R+q_{1}\lambda\left(t_{2}-t_{1}\right)$. This yields the B-estimator of the incidence rate:

$$\lambda^{B}=\frac{p_{2}-p_{1}R}{q_{1}\left(t_{2}-t_{1}\right)}$$

Using a second order approximation of equation (III-7),$p_{2}=p_{1}R+q_{1}\lambda\left(t_{2}-t_{1}\right)\left(1-\lambda\left(t_{2}-t_{1}\right)\right)$ yields the following approximation of the bias of the B-estimator:

$$E^{B}=-\lambda^{2}\left(t_{2}-t_{1}\right)$$

This shows that the B-estimator tends to underestimate the incidence rate.

Note that the expression of the standard error of the B-estimator which was given in [6] was used in this study.

**Figure S4: Mean HIV and Hallett et al. estimates.** The incidence rate at time 28 was obtained using the simulated (exact) prevalence in the age groups at time 26 and 30. Simulated (exact) mortality rates were used for the estimations. The sample size was fixed together with the number of replications (1000). (a): Mean incidence rate and Hallett's estimate [4] in the age groups at time 28, assuming equidistribution of age in each age-group considered. (b): Effects of the inclusion window on the bias and on the error bars.


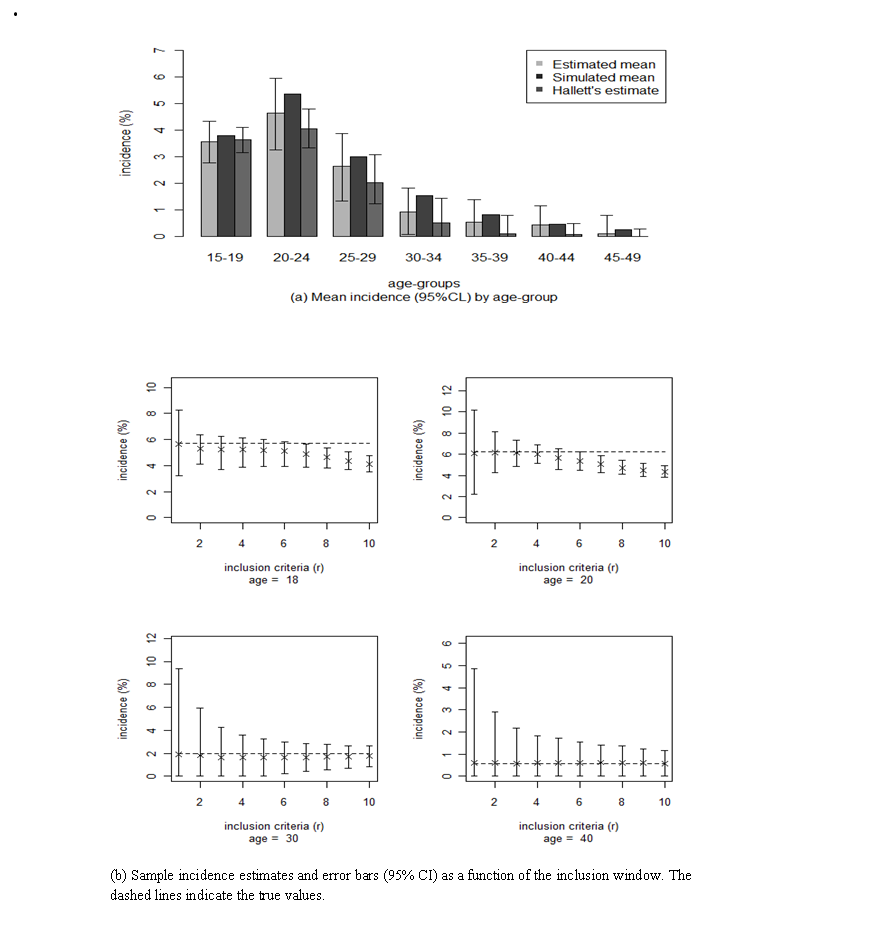


Formula (III-6) was implemented for our simulated data, with the known mortality rates. In the same time, we showed in the main text that, in the case where both the incidence and the differential mortality rates are constant, the prevalence should satisfy equation (C2), for all *t* in$\left(t_{1},t_{2}\right)$:

$$p\left(t\right)=\frac{\left(\lambda-\Delta p\left(t_{1}\right)\right)\exp\left(\left(\lambda-\Delta\right)\left(t-t_{1}\right)\right)-\lambda\left(1-p\left(t_{1}\right)\right)}{\left(\lambda-\Delta p\left(t_{1}\right)\right)\exp\left(\left(\lambda-\Delta\right)\left(t-t_{1}\right)\right)-\Delta\left(1-p\left(t_{1}\right)\right)}$$

which should be solved for $t=t_{2}$. But in general, that equation cannot be solved analytically. We investigated the possibility of obtaining a simpler formula. In effect, in the case where $T=t_{2}-t_{1}$ is not large, as it is the case in practice, for the HIV case, we can always assume that individuals of the cohort who become infected during the study do not die due to the infection. Thus, equation (C3) (or its more general version, equation (I-4)) becomes:

$$p\left(t\right)=\frac{p(t_{1})\exp\left(-\Delta\left(t-t_{1}\right)\right)+\left(1-p(t_{1})\right)\left(1-\exp\left(\lambda\left(t-t_{1}\right)\right)\right)}{1-p(t_{1})+p(t_{1})\exp\left(-\Delta\left(t-t_{1}\right)\right)}$$

For$t=t_{2}$, we obtain the G-*estimator* given by:

The above estimators were compared to the $\left(t_{1},t_{2}\right)$-estimator given in the main text by (4):

By taking the conventional age groups, if the duration between the two study is *T*=5 years, we can use the observed age-specific prevalences at time 0 and 5 to estimate the incidence rate at time 2.5, for ages 15, 20, etc. assuming that the prevalence was 0 for individuals younger than 15. To this end, either the H-estimator, the $\left(t_{1},t_{2}\right)$-estimator or the G-estimator can be used. When data is available, these can also be used to the estimate incidence rate at all ages.

Numerical results showed that Hallett's estimator and the estimator obtained from the system when we assumed that there was no possibility of seroconversion and death due to the infection between the two surveys the G-estimator gave very close estimates. This suggests that the main source of bias in the H-estimator is due to that hypothesis, for these simulations.

The contour lines for the relative error of the H-estimator, G-estimator and $\left(t_{1},t_{2}\right)$-estimator as functions of the final prevalence and the excess mortality rate are given in Figure S5. They illustrate the fact that when the excess mortality rate was different from the incidence rate, which is often the case in practice, the bias of H-estimator was negative and increased rapidly with both the excess mortality rate and the gap between the prevalence; the G-estimator underestimated the incidence rate, with a small bias for excess mortality rate close to zero; the bias of the $\left(t_{1},t_{2}\right)$-estimator was small and was reduced when the final prevalence was close to the initial prevalence. It underestimated the true value when the final prevalence was greater than the initial prevalence, with a relative error of less than 20% even for excess mortality rate and final prevalence up to 0.8, and overestimated the true value for final prevalence lower than the initial prevalence.

**Comparison of the methods using the aggregated data**

We also used the aggregated data, i.e. simulated prevalence, to estimate the incidence rate as indicated above. Figure S2 illustrates the estimated and simulated incidence rate and prevalence when the direct approach is applied to prevalences as a function of age groups of length 1. Figure S3 illustrates the estimated and simulated incidence rates and prevalence when prevalences are given as a function of the *traditional* age groups of length 5. Prevalences were first smoothed and the direct approach was then applied. Overall, the prevalence and incidence rate were underestimated for ages where the curvature in the prevalence is high, i.e. for ages in the interval 26 to 35. The incidence rate was overestimated for older ages.

**Figure S5: The relative error of some incidence estimators of in the case of birth cohort.** Contour lines for the relative error (in percentage) of the (asymptotic) estimates of the incidence rate for birth cohorts in the case where the initial prevalence is 0.1 and the time between the two surveys is *T*=5 years; background mortality rate ($\mu$) is indicated for Hallett's estimates. The errors are calculated as a function of the excess mortality rate among infected ($\Delta$), the initial prevalence ($p_{1}$) and the prevalence after *T* years ($p_{2}$). (a): $\left(t_{1},t_{2}\right)$-estimator which approximates the maximum likelihood estimates of the incidence rates; (b): G-estimator, obtained by solving the likelihood equation under the hypothesis that there is no seroconversion and death in the interval between the two surveys; (c) and (d): incidence rate estimated using the H-estimator which was proposed by Hallett et al. [4]


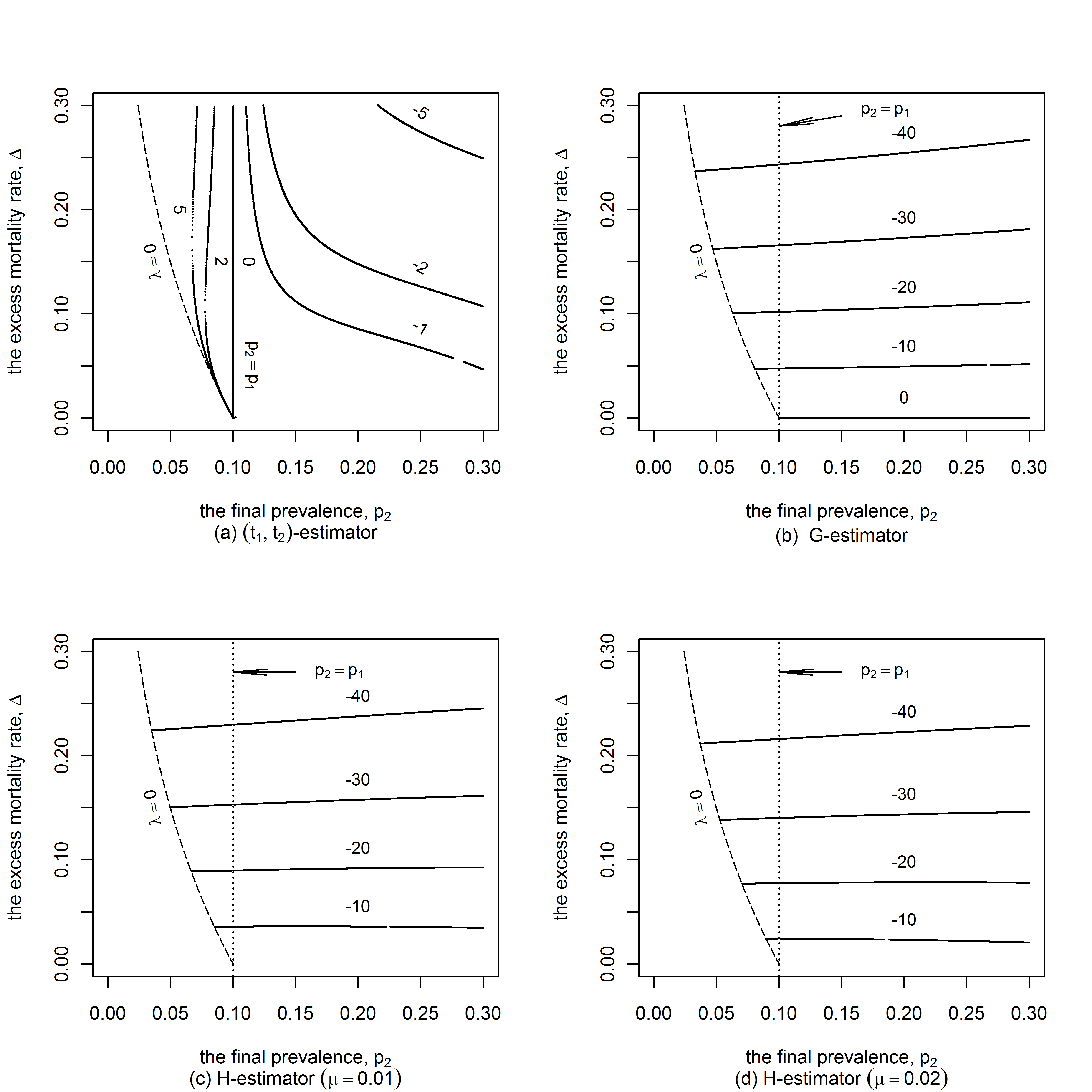


Figure S4(a) shows the simulated and estimated mean incidence rate in the 5-years age groups and the estimations obtained using the H-estimator. It shows that, for this phase, with decreasing incidence, the H-estimator underestimated the incidence rate. Figure S4(b) suggests that increasing the inclusion window may reduce the width of the confidence limits but produce a small bias. Finally, contour lines for the asymptotic bias in estimators given by (III-6), (4) and (III-8) are presented in Figure S5. The same remarks as those observed from Table 1 in the main text hold.

**IV. Accounting for migration**

We considered a non remissible infection with differential mortality in an age structured and closed population. The results obtained there can be generalised in the case where the population is subject to migration. In fact, in the general case, the dynamic of that population can be described by Figure S6. That figure indicates that individuals are recruited into the population either infected or not. We denoted the in-coming population of age *a* at time *t* by $M_{S}\left(a,t\right)$ and $M_{I}\left(a,t\right)$ among the susceptible ($S\left(a,t\right)$) and infected ($I\left(a,t\right)$) individuals, respectively. The rate of out-going (including death) for the susceptible and infected individuals was denoted $\mu_{S}\left(a,t\right)$ and$\mu_{I}(a,t)$, respectively. In a regime where $S\left(a,t\right)>0$ and $I\left(a,t\right)>0$ for all *a, t*, the system (A1) can then be re-written as follow:

where $\mu'_{S}\left(a,t\right)=\mu_{S}\left(a,t\right)-\frac{M_{S}\left(a,t\right)}{S\left(a,t\right)}$ and $\mu'_{I}\left(a,t\right)=\mu_{I}\left(a,t\right)-\frac{M_{I}\left(a,t\right)}{I\left(a,t\right)}$. Thus formula (A1)

is valid, provided $\Delta\left(a,t\right)$is defined as the difference in the net attrition rate given by .

**V. Confidence limits**

**V.1. Variances of the estimators for birth cohorts**

The asymptotic variances of the $\left(t_{1},t_{2}\right)$-estimator and the G-estimator given in section III can be obtained by the delta method. Let$T=t_{2}-t_{1}$. We have, and where $f(x,y)=\frac{x}{1-y}+\Delta x$ is differentiable in$R\times R\backslash\left\{1\right\}$, and $g(x,y)=\frac{1}{T}\left(\log\left(1-y+y\exp\left(-\Delta T\right)\right)+\log\left(\frac{1-x}{1-y}\right)\right)$is differentiable in$\left(0,1\right)\times\left(0,1\right)$. Thus we have the following approximations:

Now, and, where $\hat{p}_{1}$ and $\hat{p}_{2}$ are estimations of the prevalence at time $t_{1}$and $t_{2}$respectively. Thus

Similarly, we have:

and

where

, and

In the same way, we can estimate the asymptotic variance of the exact ML estimator given by solving equation (C2). In fact, let $\hat{\lambda}_{0}$be the ML estimator of the incidence rate. Equation (C2) can be rearranged so that $\hat{\lambda}_{0}$ is the solution of the equation$y=0$, where .Thus is the implicit solution of .

Then, the delta method gives: . Applying the implicit function theorem, we obtain:

and

Now, we have: ; and

Thus

with and

**Figure S6: Population dynamic in the case of migration.** $\lambda(a,t)$: incidence rate; $\mu_{S}(a,t)$: mortality rate for the susceptible population ($S\left(a,t\right)$); $\mu_{I}\left(a,t\right)$: mortality rate for the infected population ($I\left(a,t\right)$);$M_{S}\left(a,t\right)$:migration in the susceptible population; $M_{I}\left(a,t\right)$:migration for the infected population.


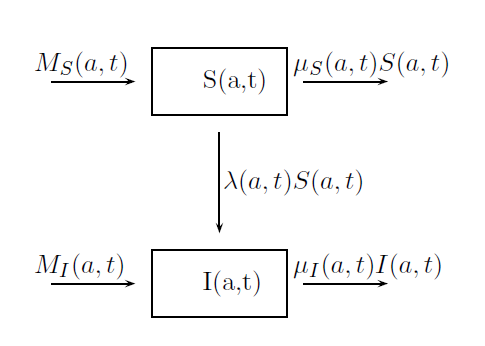


**V.2. Bootstrap confidence limits**

To determine confidence limits for estimates of incidence rates, one can follow Clayton Hills [1,7] who define the supported range for a set of possible parameters estimates as being the range of parameter values for which the deviance exceeds a predetermined critical value. Since the deviance asymptotically follows a $\text{χ}^{2}$ distribution with q degrees of freedom, where q is the number of variable parameters in the model, setting the critical value for the supported range to $\text{χ}^{2}(0.05)$ gives an approximate 95% confidence interval.

To determine the corresponding confidence limits for the derived curve, the parameters are varied over the parameter space to determine the family of curves corresponding to the supported range of the parameters. While the parameter space could be explored over a regular grid, a more efficient approach is to use the Monte Carlo Method. The maximum likelihood estimation method enables us to calculate the covariance matrix of the coefficients [8]. We can then generate normally distributed random numbers from the multivariate distribution specified by the covariance matrix. For each set of parameters we calculate the deviance and test to see if the deviance falls in the supported range.

Having generated sufficiently many runs for which the deviance falls within the supported range, typically about 1000, the maximum and minimum values of the incidence rate at each age as well as the overall incidence rate can be determined for these runs. These extreme values then give the estimated 95% confidence limits [1].

**References**

1. Williams B, Gouws E, Wilkinson D, Karim SA (2001) Estimating HIV incidence rates from age prevalence data in epidemic situations. Stat Med 20: 2003-2016.

2. Sakarovitch C, Alioum A, Ekouevi DK, Msellati P, Leroy V, et al. (2007) Estimating incidence of HIV infection in childbearing age African women using serial prevalence data from antenatal clinics. Stat Med 26: 320-335.

3. Gregson S, Donnelly CA, Parker CG, Anderson RM (1996) Demographic approaches to the estimation of incidence of HIV-1 infection among adults from age-specific prevalence data in stable endemic conditions. Aids 10: 1689-1697.

4. Hallett TB, Zaba B, Todd J, Lopman B, Mwita W, et al. (2008) Estimating incidence from prevalence in generalised HIV epidemics: methods and validation. PLoS Med 5: e80.

5. Brunet CR, Struchiner C (1999) A Non-parametric Method for the Reconstruction of Age-and Time-Dependent Incidence from the Prevalence Data of Irreversible Diseases with Differential Mortality Theoretical Population Biology 56: 76-90.

6. Brookmeyer R, Konikoff J (2011) Statistical Consideration in Determining HIV Incidence from Changes in HIV Prevalence. Statistical Communication in Infectious Diseases 3: 1-12.

7. Clayton D, Hills MH (1993) Statistical models in Epidemiology. Oxford: Oxford University press.

8. Williams BG, Dye C (1994) Maximum likelihood for parasitologists. Parasitol Today 10: 489-493.
